# Caffeine-free hawk tea lowers cholesterol by reducing free cholesterol uptake and the production of very-low-density lipoprotein

**DOI:** 10.1038/s42003-019-0396-4

**Published:** 2019-05-08

**Authors:** Juan Feng, Jian Yang, Yujun Chang, Liansheng Qiao, Honglei Dang, Kun Luo, Hongyan Guo, Yannan An, Chengmei Ma, Hong Shao, Jie Tian, Yuan Yuan, Lan Xie, Wanli Xing, Jing Cheng

**Affiliations:** 1 State Key Laboratory of Membrane Biology, School of Medicine, Tsinghua University, 100084 Beijing, China; 2 Medical Systems Biology Research Center, School of Medicine, Tsinghua University, 100084 Beijing, China; 3 National Engineering Research Center for Beijing Biochip Technology, 102206 Beijing, China; 4 State Key Laboratory Breeding Base of Dao-di Herbs, National Resource Center for Chinese Materia Medica, China Academy of Chinese Medical Sciences, 100700 Beijing, China

**Keywords:** Target identification, Gene expression analysis, Metabolic disorders

## Abstract

Medicinal plants show important therapeutic value in chronic disease treatment. However, due to their diverse ingredients and complex biological effects, the molecular mechanisms of medicinal plants are yet to be explored. By means of several high-throughput platforms, here we show hawk tea extract (HTE) inhibits Niemann–Pick C1-like 1 (NPC1L1)-mediated free cholesterol uptake, thereby inducing the transcription of low-density lipoprotein receptor (*LDLR*) downstream of the sterol response element binding protein 2 (SREBP2) pathway. Meanwhile, HTE suppresses hepatocyte nuclear factor 4α (HNF4α)-mediated transcription of microsomal triglyceride transfer protein (*MTP*) and apolipoprotein B (*APOB*), thereby decreasing the production of very-low-density lipoprotein. The catechin EGCG ((−)-epigallocatechin gallate) and the flavonoids kaempferol and quercetin are identified as the bioactive components responsible for the effects on the NPC1L1-SREBP2-LDLR axis and HNF4α-MTP/APOB axis, respectively. Overall, hawk tea works as a previously unrecognized cholesterol-lowering agent in a multi-target and multi-component manner.

## Abstract (web summary)

Juan Feng et al. show that hawk tea extract reduces cholesterol by suppressing Niemann-Pick C1 like 1-mediated free cholesterol uptake and decreasing the production of very low-density lipoprotein through hepatocyte nuclear factor 4 alpha. This study suggests a therapeutic potential of hawk tea extract for hypercholesterolemia.

## Introduction

Cardiovascular disease is the leading cause of death and disability worldwide^1^. The global number of deaths from cardiovascular diseases has increased from 12.3 million in 1990 to 17.3 million in 2013^1^. Hypercholesterolemia, especially excessive low-density lipoprotein cholesterol (LDL-c) is a major risk factor for progression of cardiovascular diseases^2^. Conventional anti-hypercholesterolemic drugs include cholesterol biosynthesis inhibitors (e.g., Statins)^3^ and cholesterol absorption inhibitors (e.g., Ezetimibe)^4^. Emerging cholesterol-lowering drugs in recent years include proprotein convertase subtilisin/kexin type 9 monoclonal antibodies (e.g., Evolocumab)^5^, microsomal triglyceride transfer protein (MTP) inhibitors (e.g., Lomitapide)^6^, and apolipoprotein B (*APOB*) antisense oligonucleotides (e.g., Mipomersen)^7^. However, the side effects of these drugs cannot be ignored, such as Statin-induced muscle pain or Lomitapide-induced elevated liver transaminases^8^. In many cases, combinations of two or more kinds of drugs are necessary to achieve better efficacy.

Medicinal plants usually contain multiple components and have the potential to target combinatorial pathways with less side effects^9^, thus providing an alternative for more effective and safer control of hypercholesterolemia. For example, as a popular beverage in the world, the *Camellia* tea green tea is well documented to exhibit hypolipidemic and hypoglycemic properties^10^. However, the high levels of caffeine in green tea may cause side effects like sleep deprivation, anxiety, elevated blood pressure, and so on, making it unsuitable for specific populations such as pregnant women and older cardiovascular disease patients^11^.

There is a class of tea that derives from non-*Camellia* natural plants and is processed and consumed in similar ways as traditional *Camellia* teas^12^. As a non-*Camellia* tea, hawk tea, which is made from buds or leaves of *Litsea coreana* Levl. var. lanuginose (a medicinal plant recorded in the Xinhua Materia Medica Outline), is very popular in southwestern China. For many years, it has been used as a folk medicine to treat gastrosis, hepatitis, and inflammatory diseases^13^. Modern pharmacological studies demonstrate that hawk tea has protective effects against liver fibrosis, hypercholesterolemia, hyperglycemia, and inflammatory diseases^14^. However, the molecular mechanism and bioactive components remain unclear. Here we show that hawk tea works by targeting two key axes in cholesterol metabolism. On the one hand, hawk tea extract (HTE) inhibits Niemann–Pick C1-like 1 (NPC1L1)-mediated hepatic-free cholesterol uptake, thereby inducing sterol response element binding protein 2 (SREBP2)-mediated low-density lipoprotein receptor (LDLR) expression. On the other hand, HTE inhibits MTP- and APOB-mediated very-low-density lipoprotein (VLDL) production by suppressing hepatocyte nuclear factor 4α (HNF4α). The effects of HTE on NPC1L1-SREBP2-LDLR axis and HNF4α-MTP/APOB axis can be attributed to the catechin EGCG ((−)-epigallocatechin gallate) and the non-catechin flavonoids kaempferol and quercetin, respectively.

## Results

### HTE ameliorates dyslipidemias and disordered gut microbiota

**Fig. 1:**
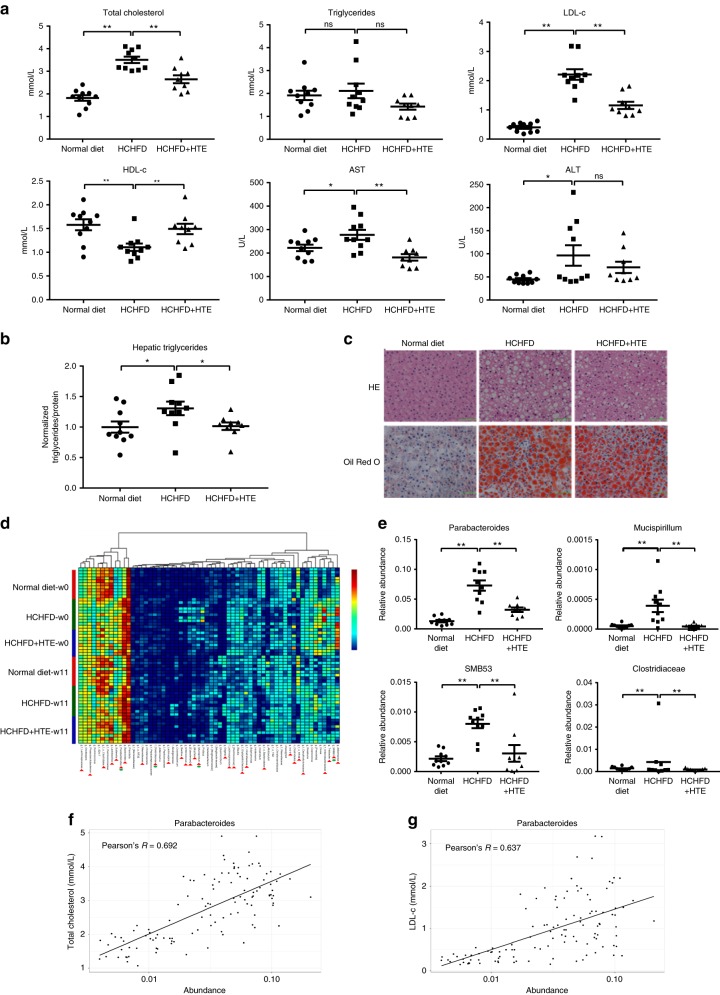
Hawk tea extract (HTE) ameliorates dyslipidemias and disordered gut microbiota. **a** Serum levels of total cholesterol, triglycerides, low-density lipoprotein cholesterol (LDL-c), high-density lipoprotein cholesterol (HDL-c), aspartate aminotransferase (AST), and alanine aminotransferase (ALT) in rats of the normal diet group (*n* = 10), high-cholesterol and high-fat diet (HCHFD) group (*n* = 10), and HCHFD + HTE group (*n* = 9). **b** Hepatic triglycerides levels in rats of three groups. **c** Representative hematoxylin–eosin (HE) and Oil Red O staining analysis for liver specimens. Scale bar = 100 μm. **d** Heatmap of 60 most predictive genera in rats of three groups. w0: the beginning time point of HTE intervention; w11: the 11th week after HTE intervention. Red triangles: microbes modulated by HCHFD at w0 (vs. the normal diet group); green circles: microbes modulated by HTE intervention at w11 (vs. the HCHFD model group). The color of each spot in the heatmap corresponds to the normalized and log-transformed relative abundance of each genus in samples by a gradient of color from blue (low abundance) to red (high abundance). The taxonomic assignment of each genus was determined by Ribosomal Database Project classifier and the genus name and sample group name was labeled in the graph. **e** The relative abundance of four typical genera modulated by HTE. **f** Pearson’s correlation dot plots between *Parabacteroides* and serum total cholesterol and **g** LDL-c levels, respectively. The filled squares, circles and triangles indicate individual data points of normal diet group, HCHFD group, and HCHFD+HTE group, respectively. Data are shown in mean ± SEM. For **a**, **b**, statistical analyses were conducted using non-paired Student’s *t* test. For **d**, **e**, statistical analyses were conducted using one-way analysis of variance (ANOVA) followed by Tukey’s honest significant difference test. *p < 0.05; **p < 0.01; ns: not significant

A high-cholesterol and high-fat diet (HCHFD)-induced hypercholesterolemic rat model was used to evaluate the cholesterol-lowering effects of HTE. The HCHFD model group exhibited elevated serum total cholesterol and LDL-c levels compared to the normal diet group (Fig. 1a). HTE dramatically lowered total cholesterol and LDL-c levels and increased the high-density lipoprotein cholesterol (HDL-c) level in the blood, but did not alter serum triglycerides (Fig. 1a). The HCHFD increased the levels of serum transaminases, aspartate aminotransferase (AST), and alanine aminotransferase (ALT) (Fig. 1a). HTE treatment reversed serum AST to normal levels and also yielded a decrease trend in ALT levels (Fig. 1a), suggesting that HTE has a protective effect on the compromised liver function caused by HCHFD.

In the liver, HTE reversed the high triglycerides levels induced by HCHFD (Fig. 1b). However, there was no significant difference in the hepatic cholesterol levels between the HCHFD and the HCHFD + HTE group (*p* > 0.05, Supplementary Fig. 1a). Histological analysis showed that HTE relieved cell swelling and vacuolation (representative results are shown in Fig. 1c and the complete staining panel is available in Supplementary Fig. 1b). Oil Red O staining demonstrated that the number and size of lipid droplets in the livers of most HTE-treated rats decreased compared to the HCHFD group (Fig. 1c and Supplementary Fig. 1c), consistent with the decrease in liver triglycerides (Fig. 1b).

The decreases in AST and ALT in the HCHFD+HTE group indicated that HTE has no hepatotoxicity and even protects liver from steatosis. Further, we examined the nephrotoxicity of HTE by analyzing serum levels of blood urea nitrogen (BUN) and creatinine (CREA). BUN and CREA after the administration of HTE were in normal ranges, indicating that HTE has no nephrotoxicity (Supplementary Fig. 1d). Overall, HTE effectively and safely ameliorated HCHFD-induced hypercholesterolemia and liver steatosis in rats.

Accumulating evidence suggests that the gut microbiome plays a critical role in the development of human diseases, such as inflammatory diseases and metabolic disorders, and is a promising drug target for disease treatment^15^. To investigate changes in gut microbiota composition after HTE administration, 16S ribosomal DNA (rDNA) amplicon sequencing was performed using the Illumina MiSeq platform. We obtained an average of 70,942 sequence reads per sample and identified 235 genera in all samples. Among the 235 genera, 60 were selected for taxon-based analysis of gut microbiota composition in the normal diet, HCHFD, and HCHFD + HTE groups (see the Methods section for the selection criteria). HCHFD feeding altered the abundance of 30 genera (Fig. 1d, w0: HCHFD vs. normal diet group, indicated with red triangles). Among them, the abundance of *Parabacteroides*, *SMB53*, *Mucispirillum*, and *Clostridiaceae* spp. (Fig. 1d, w11: HCHFD+HTE vs. HCHFD group, indicated with green circles) was decreased by HTE (Fig. 1d, e). These four microbes are all positively associated with obesity or type 2 diabetes and can be modulated by western medicine (e.g., Metformin) or traditional Chinese medicine (e.g., *Coptidis chinensis* and *Ganoderma lucidum*)^15–18^. The modulation of HTE on their abundance indicated that HTE partially improves HCHFD-induced changes in gut microbial communities.

Next, an association study based on partial least-squares regression was performed between the microbial genera abundance and serum cholesterol levels. Notably, *Parabacteroides*, which was dramatically enriched in the HCHFD group and reduced by HTE, displayed the highest correlation with serum total cholesterol and LDL-c levels (Supplementary Tables 1, 2 and Fig. 1f, g), suggesting that *Parabacteroides* is a potential target of HTE and might contribute to its cholesterol-lowering effects in rats.

### HTE alters pathways related to lipid metabolism in vivo

To identify the global transcriptome changes associated with HTE in vivo, we performed RNA-sequencing (RNA-seq) of liver samples obtained from the normal diet, HCHFD, and HCHFD + HTE groups (GEO: GSE125084). A total of 3070 differentially expressed genes (cutoff: fold change ≥2 and *p* value ≤0.05) were identified between the HCHFD/normal diet groups (1500 up-regulated and 1570 down-regulated), and 1004 were identified between the HCHFD + HTE/HCHFD groups (516 up-regulated and 488 down-regulated).

**Fig. 2:**
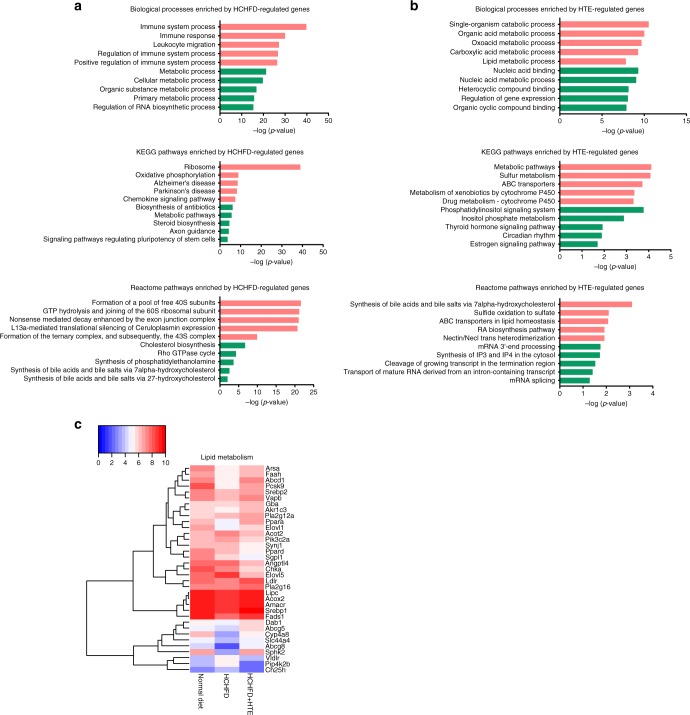
Hawk tea extract (HTE) alters pathways related to lipid metabolism in vivo. **a** –Log_10_(*p* value) of the top five enriched biological processes, KEGG pathways, Reactome pathways by high-cholesterol and high-fat diet (HCHFD)-regulated genes (vs. the normal diet group). Red: up-regulated; green: down-regulated. **b** –Log_10_(*p* value) of the top five enriched biological processes, KEGG pathways, Reactome pathways by HTE-regulated genes (vs. the HCHFD group). Red: up-regulated; green: down-regulated. **c** Heatmap of differential expression of genes implicated in lipid metabolism. The color of each spot in the heatmap corresponds to log-transformed average relative expression levels of each gene in three samples by a gradient of color from blue (low expression level) to red (high expression level)

Genes that were up-regulated by the HCHFD treatment were mainly involved in immune processes and ribosome-related pathways, while down-regulated genes resulted in the enrichment of metabolic processes, the cholesterol biosynthesis pathway, and the bile acid biosynthesis pathway (Fig. 2a). These results indicated that the HCHFD strongly interfered with the metabolism of rats.

Interestingly, the lipid metabolic processes, metabolic pathway, and bile acid biosynthesis pathway were also enriched by genes up-regulated by HTE treatment in the disease model (Fig. 2b), indicating that HTE can somehow reverse changes of lipid metabolism induced by HCHFD supplementation. Heatmap of the differential expression of genes implicated in lipid metabolism (e.g., *Srebp1*, *Srebp2*, and *Ldlr*) among the three groups also suggested the reversing effect of HTE against HCHFD (Fig. 2c).

### HTE alters pathways related to lipid metabolism in vitro

To further explore the molecular mechanism underlying the cholesterol-lowering function of HTE, we used an immortalized hepatocyte cell line HepG2 for subsequent studies. A microarray-based high-throughput gene expression profiling of HepG2 cells treated with HTE was performed (GEO: GSE117583). A total of 1889 differentially expressed genes (1080 up-regulated and 809 down-regulated; cutoff: 2-fold) were identified after HTE treatment at a dose of 300 μg mL^−1^ for 24 h. The concentration of tea extracts in cell lines reported in the literature is usually in the range of 100–300 μg mL^−1^ (refs ^19–21^). We chose a relatively high dosage (300 μg mL^−1^) for the microarray analysis to achieve an adequate disturbance of the genome-wide gene expression for further analysis. At this dosage, cell viability was affected slightly, approximately by 30% (Supplementary Fig. 2). Considering that cell viability >40% is acceptable for microarray analysis^22^, the nearly 70% cell viability under this dosage is tolerable for cells.

**Fig. 3:**
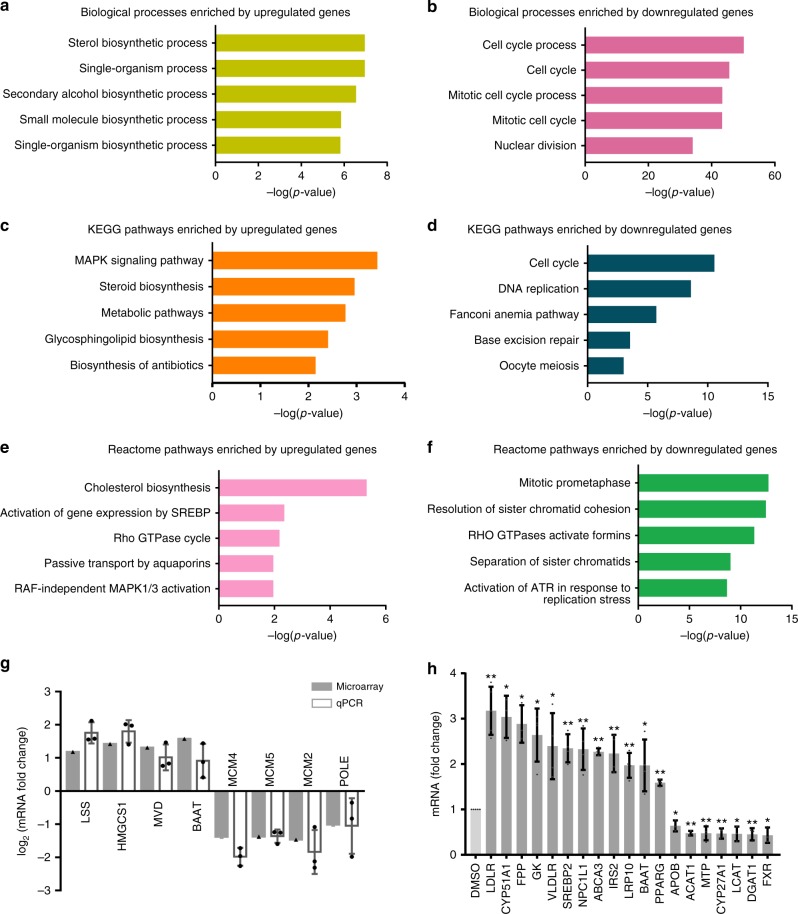
Hawk tea extract (HTE) alters pathways related to lipid metabolism in vitro. **a** –Log_10_(*p* value) of the top five enriched biological processes by the up-regulated and **b** the down-regulated genes, respectively. **c** –Log_10_(*p* value) of the top five enriched KEGG pathways by the up-regulated and **d** the down-regulated genes, respectively. **e** –Log_10_(*p* value) of the top five enriched Reactome pathways by the up-regulated and **f** the down-regulated genes, respectively. **g** Quantitative real-time PCR (qRT-PCR) validation for the expression of representative genes in steroid biosynthesis pathway (*LSS* and *HMGCS1*), metabolic pathways (*MVD* and *BAAT*), cell cycle signaling pathway (*MCM4* and *MCM5*), and DNA replication pathway (*MCM2* and *POLE*) (*n* = 3). **h** qRT-PCR analysis of a set of genes related to cholesterol metabolism under HTE (300 μg mL^−1^) treatment in HepG2 cells (*n* ≥ 3). Data are shown in mean ± SD. Statistical analyses were conducted using paired Student’s *t* test. *p < 0.05 vs. dimethyl sulfoxide (DMSO)-treated group; **p < 0.01 vs. DMSO-treated group. The filled triangles and filled circles indicate data of an individual independent microarray analysis and qPCR experiment, respectively

Gene Ontology and pathway analysis revealed that the down-regulated genes were highly enriched in pathways such as DNA replication and cell cycle. The up-regulated genes were highly enriched in pathways related to cholesterol metabolism (e.g., the steroid biosynthesis, cholesterol biosynthesis, and activation of gene expression by SREBP) (Fig. 3a–f), similar to what we found in rats. The expression of several representative genes (steroid biosynthesis pathway: *LSS* and *HMGCS1*; metabolic pathways: *MVD* and *BAAT*; cell cycle pathway: *MCM4* and *MCM5*; and DNA replication pathway: *MCM2* and *POLE*) was confirmed by three independent quantitative real-time PCR (qRT-PCR) experiments, and the results agreed well with the microarray analyses (Fig. 3g).

Considering that HTE affects pathways related to cholesterol metabolism, we assessed the expression of more key genes, such as *LDLR* and *VLDLR*, which mediate the uptake of LDL and VLDL; *FPP* and *CYP51A1*, encoding two enzymes in the steroid biosynthesis pathway; *MTP* and *APOB*, participating in the production of VLDL; *DGAT1*, catalyzing the biosynthesis of triglycerides; and *FXR*, a nuclear receptor regulating bile acid synthesis and hepatic triglycerides levels. The expression of all of these genes (*LDLR*, *CYP51A1*, *FPP*, *GK*, *VLDLR*, *SREBP2*, *NPC1L1*, *ABCA3*, *IRS2*, *LRP10*, *BAAT*, *PPARγ*, *APOB*, *ACAT1*, *MTP*, *CYP27A1*, *LCAT*, *DGAT1*, and *FXR*) was altered in response to HTE (Fig. 3h), implying that HTE modulates cholesterol metabolism by interfering the expression of multiple genes.

### HTE promotes SREBP2-mediated *LDLR* transcription

**Fig. 4:**
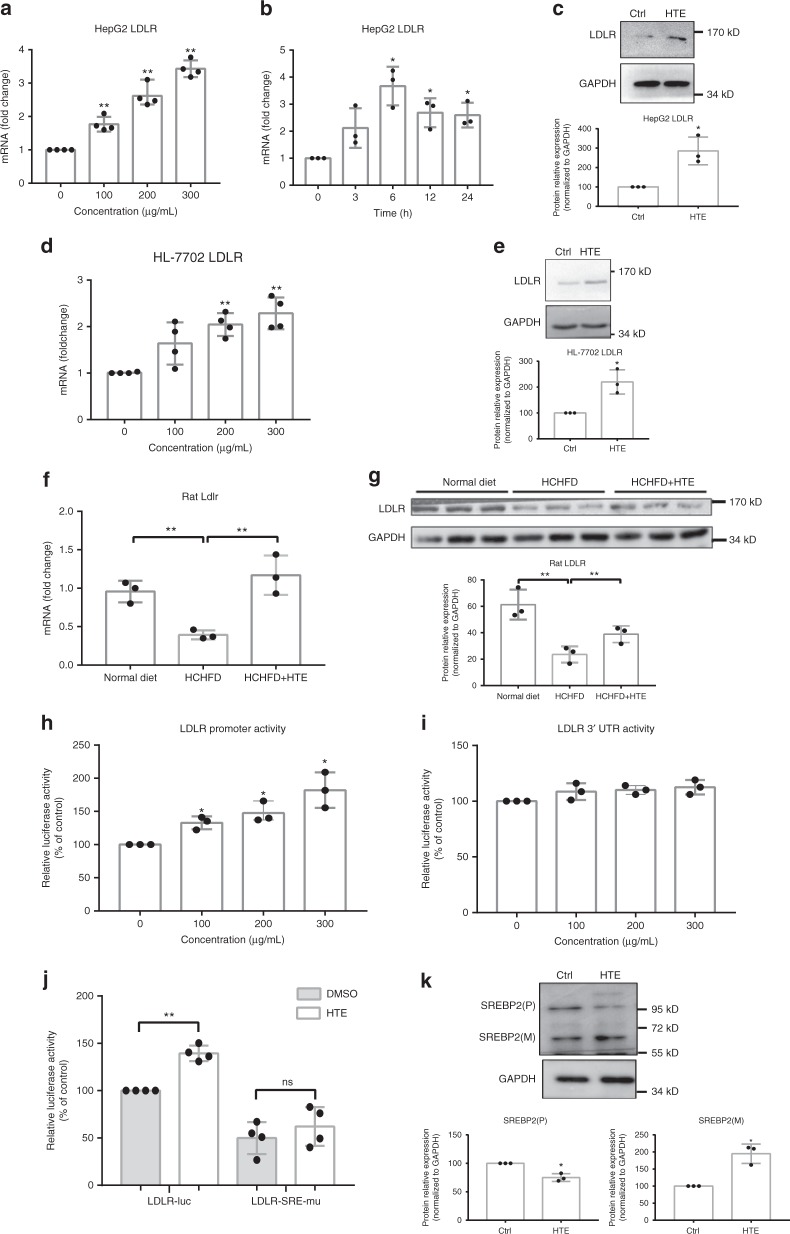
Hawk tea extract (HTE) promotes sterol response element binding protein 2 (SREBP2)-mediated low-density lipoprotein receptor (*LDLR*) transcription. **a** Dose–response profiles of *LDLR* expression under HTE treatment in HepG2 cells (*n* = 4). **b** Time course of *LDLR* expression. **c** Representative Western blot analysis of LDLR and quantitative analysis of LDLR protein under HTE (200 μg mL^−1^) treatment in HepG2 cells (*n* = 3). Original Western blot images are shown as the Supplementary Fig. 9. **d** Dose–response profiles of *LDLR* expression under HTE treatment in HL-7702 cells (*n* = 4). **e** Representative Western blot analysis of LDLR and quantitative analysis of LDLR protein under HTE treatment in HL-7702 cells (*n* = 3). Original Western blot images are shown as the Supplementary Fig. 10. **f** Quantitative real-time PCR (qRT-PCR) analysis of liver *Ldlr* gene in rats of three groups (*n* = 3). **g** Representative Western blot analysis of LDLR and quantitative analysis of LDLR protein in rat livers of three groups (*n* = 3). Original Western blot images are shown as the Supplementary Fig. 11. **h** Dose–response profiles of luciferase activity of the *LDLR* promoter and **i** *LDLR* 3′-untranslated region (3′-UTR) under HTE treatment in HeLa cells (*n* = 3). **j** Luciferase activity of sterol response element (SRE)-mutated pGL3-LDLR under HTE (200 μg mL^−1^) treatment in HeLa cells (*n* = 4). **k** Representative Western blot analysis of precursor (P) and mature (M) form of SREBP2 and quantitative analysis of the proteins under HTE (200 μg mL^−1^) treatment in HepG2 cells (*n* = 3). Original Western blot images are shown as the Supplementary Fig. 12. Data are shown in mean ± SD. For **a**–**e**, **h**–**k**, statistical analyses were conducted using paired Student’s *t* test. For **f**, **g**, statistical analyses were conducted using non-paired Student’s *t* test. *p < 0.05; **p < 0.01; ns, not significant. Filled circles indicate the individual data points of each independent experiments under the indicated treatment conditions

LDLR plays a critical role in cholesterol homeostasis and mediates the clearance of LDL-c in blood^23^. We noticed it as a highly up-regulated gene of HTE both in rat livers (Fig. 2c) and HepG2 cells (Fig. 3h), based on the transcriptome analysis. By the qRT-PCR in HepG2 cells, we found that the induction of *LDLR* by HTE was dose-dependent, reaching a maximum of ~3.4-fold at 300 μg mL^−1^ (Fig. 4a). For time-course experiments, *LDLR* messenger RNA (mRNA) was up-regulated very soon after treatment and the levels peaked at 6 h (~3.6-fold) and then declined to nearly 2.6-fold at 24 h (Fig. 4b). The up-regulation of mRNA expression did lead to an increase in LDLR protein in HepG2 cells (Fig. 4c). The effect of HTE on LDLR expression was further confirmed in another human hepatocyte cell line, HL-7702 (Fig. 4d, e). We also verified the effect of HTE on LDLR expression in vivo. Consistently, an increase in LDLR expression at both the mRNA and protein levels was observed in HCHFD+HTE group compared to the HCHFD group (Fig. 4f, g).

Next, luciferase reporter assays were performed in HeLa cells to investigate how HTE affects the promoter and 3′-untranslated region (3′-UTR) activity of *LDLR*. HTE enhanced *LDLR* promoter (−1100 to +187 bp) activity in a dose-dependent manner (Fig. 4h), suggesting that HTE regulates *LDLR* expression at the transcriptional level. Certain drugs such as berberine has been reported to up-regulate *LDLR* expression at the post-transcriptional level through ERK (extracellular signal regulated kinase)-mediated 3′-UTR stabilization^24^. However, HTE had no effect on the activity of the *LDLR* 3′-UTR (Fig. 4i) and did not increase the phosphorylation of ERK (Supplementary Fig. 3), implying that the regulation of *LDLR* expression by HTE is distinct from that of berberine.

Regulation of *LDLR* at the transcriptional level dominantly relies on the sterol response element (SRE), which is the binding site of SREBP2, and can be modulated by drugs like Statins^25^. Based on our Reactome pathway analysis of HTE-treated HepG2 cells, activation of gene expression by SREBP pathway was highly enriched by HTE up-regulatory genes (Fig. 3g). We constructed plasmids harboring the *LDLR* promoter with the SRE motif mutated (ATCACCCC→ATCATTCC), and luciferase reporter assays in HeLa cells were performed. The stimulatory effect of HTE on the *LDLR* promoter was abolished by mutation of the SRE motif (Fig. 4j), supporting the notion that HTE-mediated elevation of *LDLR* mRNA levels relies on the SRE motif. Western blot analysis in HepG2 cells showed that the precursor form of SREBP2 was decreased, and the mature form of SREBP2 was increased by HTE treatment (Fig. 4k), confirming the activation of SREBP2 pathway by HTE.

### HTE inhibits NPC1L1-mediated free cholesterol uptake

As shown above, the SREBP2 pathway was activated by HTE, resulting in an increase in LDLR expression. Maturation of SREBP2 is activated under sterol-depleted conditions, but it remains inactive under sterol-replete states^26^. Here, we asked if the activation of SREBP2 by HTE treatment is a consequence of sterol deficiency.

**Fig. 5:**
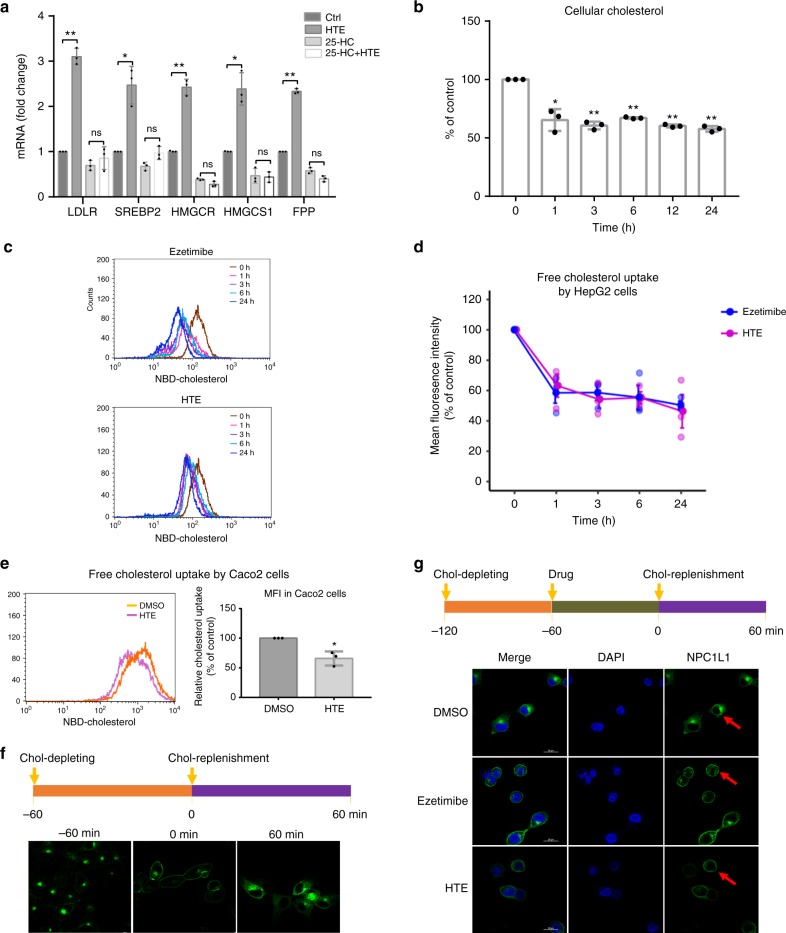
Hawk tea extract (HTE) inhibits Niemann–Pick C1-like 1 (NPC1L1)-mediated free cholesterol uptake. **a** The effect of 25-hydroxycholesterol (25-HC) (0.5 μg mL^−1^) on the stimulatory effects of HTE (200 μg mL^−1^) on low-density lipoprotein receptor (LDLR) and other sterol response element binding protein 2 (SREBP2) downstream genes (*SREBP2*, *HMGCS1*, *HMGCR*, and *FPP*) in HepG2 cells (*n* = 3). **b** Time course of HTE (200 μg mL^−1^) effect on cellular total cholesterol level in HepG2 cells (*n* = 3). **c** Representative flow cytometry analysis of NBD (22-(*N*-(7-nitrobenz-2-oxa-1, 3-diazol-4-yl)-labeled free cholesterol uptake and **d** quantitative analysis of free cholesterol uptake by HepG2 cells under HTE (200 μg mL^−1^) or Ezetimibe (50 μΜ) treatment for different time durations (*n* = 3). The mean fluorescence intensity (MFI) at different time points indicates the amount of NBD-labeled free cholesterol uptake by HepG2 cells. NBD-cholesterol was delivered to cells in ethanol. The MFI of untreated cells was set as 100%. **e** Representative flow cytometry analysis of NBD-labeled free cholesterol uptake under HTE (200 µg mL^−1^) treatment for 1 h and quantitative analysis of free cholesterol uptake by Caco2 cells (*n* = 3). **f** Cholesterol-regulated NPC1L1 translocation between the plasma membrane and endocytic recycling compartment (ERC) in CRL1601/NPC1L1-EGFP cells. The green fluorescence signals indicate the distribution of NPC1L1 protein. **g** The effect of HTE on the translocation of NPC1L1 from plasma membrane to ERC in CRL1601/NPC1L1-EGFP cells. Cells were treated as shown in the diagram (HTE: 100 μg mL^−1^; Ezetimibe: 50 μΜ). Scale bar = 20 μm. The green fluorescence signals indicate the distribution of NPC1L1 protein and the arrows indicate the localization of plasma membrane. Data are shown in mean ± SD. Statistical analyses were conducted using paired Student’s *t* test. *p < 0.05; **p < 0.01; ns: not significant. Filled circles indicate the individual data points of each independent experiments under the indicated treatment conditions

We assessed whether 25-hydroxycholesterol (25-HC), a sterol that inhibits SREBP2 cleavage^26^, affects the ability of HTE to up-regulate *LDLR* expression. First, we demonstrated that *LDLR* and other SREBP2-downstream genes (e.g., *HMGCR* and *FPP*) were down-regulated by 25-HC in a dose-dependent manner, and concentrations higher than 0.5 μg mL^−1^ (i.e., 0.5, 1, 2, and 10 μg mL^−1^) were all effective to achieve significant inhibition of these genes (*p* < 0.05, Supplementary Fig. 4). Furthermore, we found HTE-induced up-regulation of *LDLR* and other SREBP2-regulated genes (*SREBP2*, *HMGCR*, *HMGCS1*, and *FPP*) can be abolished by the minimal effective dose of 0.5 μg mL^−1^ 25-HC (Fig. 5a), supporting the view that activation of SREBP2 is a feedback consequence of sterol deficiency caused by HTE treatment.

Thereafter, we examined the cellular cholesterol levels in HepG2 cells upon exposure to HTE. The content of total cholesterol in HepG2 cells was indeed decreased by HTE treatment, and this decrease can be observed as early as 1 h (Fig. 5b). The decrease in total cellular cholesterol could be ascribed to three possible mechanisms: an inhibition of cholesterol synthesis, an increase of cholesterol efflux, or an inhibition of free cholesterol uptake. To evaluate the effects of HTE on cholesterol biosynthesis, we examined the precursors of cholesterol synthesis in HepG2 cells, including lathosterol and desmosterol, which are commonly used as markers of cholesterol synthesis^27^, with Lovastatin as a positive control. Cellular lathosterol and desmosterol remained unchanged under HTE treatment but were decreased by Lovastatin (Supplementary Fig. 4a), indicating that HTE lowers cellular cholesterol but not via inhibiting cholesterol biosynthesis. The rate of cholesterol efflux was evaluated by NBD (22-(*N*-(7-nitrobenz-2-oxa-1, 3-diazol-4-yl)-labeled cholesterol assays and was also not altered by HTE treatment (Supplementary Fig. 5b).

Next, we tested the effect of HTE on free cholesterol uptake by flow cytometry, using Ezetimibe as a positive control. HepG2 cells treated with HTE exhibited decreased free cholesterol uptake capacity (by ~36.83%) within 1 h compared to vehicle-treated cells (Fig. 5c, d), with a similar effect to Ezetimibe (by ~41.52%, Fig. 5d). This inhibitory effect was further exacerbated over time (Fig. 5d). In addition, the inhibition of free cholesterol uptake by HTE can also be reproduced in Caco2 cells (Fig. 5e), an in vitro model of small intestinal absorptive enterocytes. These data demonstrated that the sterol deficiency caused by HTE treatment is a consequence of impaired free cholesterol uptake.

NPC1L1 plays a vital role in cholesterol absorption and recycles between the cellular endocytic recycling compartments (ERCs) and plasma membrane according to cholesterol content^28^. NPC1L1 distributes on the brush border membrane of intestinal enterocytes and the canalicular membrane of hepatocytes in humans, mediating the uptake of dietary cholesterol and biliary cholesterol, respectively^29^. Ge et al.^30^ report that after the knockdown of NPC1L1 in HL-7702, the uptake of free cholesterol by human liver cells was decreased by roughly 60%. Ezetimibe inhibits free cholesterol uptake by blocking the endocytosis of NPC1L1 in both intestines and livers^31^. Here we questioned whether NPC1L1 is involved in the decreased free cholesterol uptake caused by HTE. Considering that HTE does not reduce the expression of *NPC1L1* in HepG2 cells (Fig. 3h) or Caco2 cells (Supplementary Fig. 5c), we questioned whether HTE works by disturbing the trafficking of NPC1L1 in a manner similar to Ezetimibe. To address this issue, NPC1L1 localization studies were conducted in CRL1601 rat hepatoma cells stably expressing NPC1L1-EGFP fusion protein (referred to CRL1601/NPC1L1-EGFP cells). NPC1L1 mainly localized to the perinuclear ERC region in normal cholesterol-rich medium (Fig. 5f, −60 min), translocated to the plasma membrane after 1 h of cholesterol depletion (Fig. 5f, 0 min), and returned to the perinuclear region again after 1 h of cholesterol replenishment (Fig. 5f, 60 min). To study the effect of drugs on the endocytosis of NPC1L1 from the plasma membrane to ERC, cells were pre-treated with Ezetimibe or HTE for 1 h before cholesterol replenishment. Ezetimibe did inhibit the endocytosis of NPC1L1 (Fig. 5g). Intriguingly, the endocytosis of NPC1L1 induced by cholesterol replenishment was also blocked by HTE treatment (Fig. 5g). Therefore, HTE inhibited free cholesterol uptake by disturbing the endocytosis of NPC1L1 in a manner similar to Ezetimibe.

Taken together, one mechanism by which HTE lowers serum cholesterol is that HTE inhibits NPC1L1-mediated free cholesterol uptake, leading to a decrease in intracellular cholesterol levels, followed by the activation of the SREBP2 pathway and up-regulation of LDLR expression. This subsequently promotes LDL-c clearance in the blood.

### HTE inhibits HNF4α-mediated transcription of *MTP* and *APOB*

**Fig. 6:**
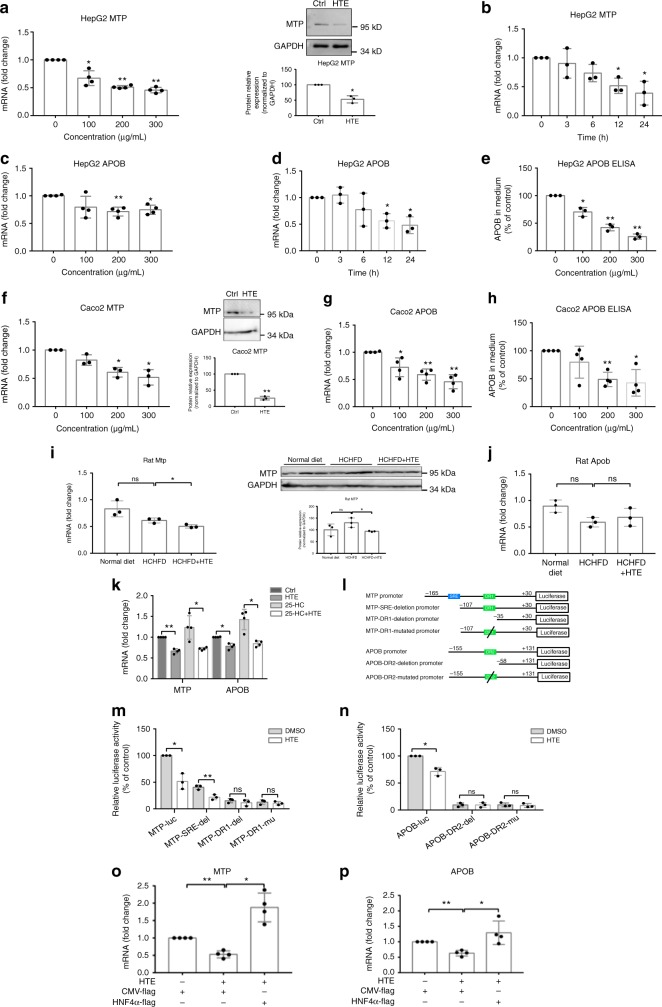
Hawk tea extract (HTE) inhibits hepatocyte nuclear factor 4α (HNF4α)-mediated transcription of microsomal triglyceride transfer protein (MTP) and apolipoprotein B (APOB). **a** Dose–response profile of *MTP* expression (*n* = 4), representative Western blot analysis of MTP and quantitative analysis of MTP protein in HepG2 cells (*n* = 3). Original Western blot images are shown as the Supplementary Fig. 13. **b** Time course of *MTP* expression under HTE (200 μg mL^−1^) treatment in HepG2 cells (*n* = 3). **c** Dose–response profile of *APOB* expression in HepG2 cells (*n* = 4). **d** Time course of *APOB* expression under HTE (200 μg mL^−1^) treatment in HepG2 cells (*n* = 3). **e** APOB secretion under HTE treatment in HepG2 cells (*n* = 3). **f** Dose–response profile of *MTP* expression, representative Western blot analysis of MTP and quantitative analysis of MTP protein under HTE (200 μg mL^−1^) treatment in Caco2 cells (*n* = 3). Original Western blot images are shown as the Supplementary Fig. 14. **g** Dose–response profile of *APOB* expression in Caco2 cells (*n* = 4). **h** APOB secretion under HTE treatment in Caco2 cells (*n* = 4). **i** Quantitative real-time PCR (qRT-PCR) of *Mtp*, representative Western blot analysis of MTP and quantitative analysis of MTP protein in rat livers of three groups (*n* = 3). Original Western blot images are shown as the Supplementary Fig. 15. **j** qRT-PCR of *Apob* in rat livers of three groups (*n* = 3). **k** The effect of 25-hydroxycholesterol (25-HC) (10 μg mL^−1^) on the repression effects of HTE (200 μg mL^−1^) on *MTP* and *APOB* in HepG2 cells (*n* = 4). **l** Schematic diagram of different reporter constructs of *MTP* and *APOB* promoter inserted in the pGL3-enhancer vector. **m** Luciferase activity of different constructs of *MTP* promoter and **n** *APOB* promoter in exposure to HTE (200 μg mL^−1^) in HeLa cells (*n* = 4). **o** qRT-PCR analysis of *MTP* and **p** *APOB* in response to HTE (200 μg mL^−1^) with or without HNF4α overexpression in HeLa cells (*n* = 3). Data are shown in mean ± SD. For **a**–**h**, **k**, **m**–**p**, statistical analyses were conducted using paired Student’s *t* test. For **i**, **j**, statistical analyses were conducted using non-paired Student’s *t* test. *p < 0.05; **p < 0.01; ns: not significant. Filled circles indicate the individual data points of each independent experiments under the indicated treatment conditions

There is one group of LDLR negative familial hypercholesterolemia patients that have markedly reduced responses to drugs requiring increased LDLR activity to mediate their effects, for example, Statins^7^. For these individuals, an alternative solution is to block the production of LDL. The LDL particles in circulation are derived from VLDL, which is packaged in the liver through MTP to load lipids onto newly synthesized APOB and secreted into the blood^32^. We noticed the down-regulation of both *MTP* and *APOB* genes in the qRT-PCR analysis of cholesterol metabolism-related genes (Fig. 3h), implicating that HTE may also disturb the process of VLDL production. Thus, we further verified the dose and time responses of these two genes under HTE treatment based on qRT-PCR. The inhibitory effects of HTE on *MTP* and *APOB* both enlarged over dose and time. The down-regulation of *MTP* by HTE reached a maximum of ~55% at 300 μg mL^−1^ HTE at 24 h (Fig. 6a, b), and the maximal decrease of *APOB* was achieved as ~30% at 300 μg mL^−1^ HTE at 24 h (Fig. 6c, d). Consequently, the protein abundance of MTP was decreased by HTE treatment in HepG2 cells (Fig. 6a), and the secretion of APOB-containing particles in HepG2 cells was indeed dose-dependently suppressed by HTE treatment, as confirmed by enzyme-linked immunosorbent assay (ELISA) (Fig. 6e). In addition, the inhibitory effects of HTE on MTP and APOB expression were also confirmed in intestine-derived Caco2 cells. The mRNA abundance of *MTP* and *APOB* in Caco2 cells was decreased by HTE in a dose-dependent manner (Fig. 6f, g), and the protein abundance of MTP as well as the secretion of APOB-containing lipoproteins by Caco2 cells was also decreased upon HTE treatment (Fig. 6f, h).

Then, we also examined the expression of MTP and APOB in rat livers. MTP was decreased by HTE in rat livers at both the mRNA and protein levels (Fig. 6i), consistent with the results in HepG2 cells. However, the expression of *Apob* was not altered by HTE in rat livers (Fig. 6j), inconsistent with that in HepG2 cells. Dixon et al.^33^ have reviewed the differential regulation of APOB in rat hepatocytes compared to cultured HepG2 cells. The inconsistency in APOB regulation in vitro and in vivo are commonly observed in many studies, possibly due to the differences between immortalized cell models and in vivo models^33^. Although the down-regulation of MTP and APOB in cultured cells by HTE is not completely applicable to rats, further study of the molecular mechanism by which HTE down-regulates these two genes in HepG2 cells will help to better delineate the mechanism of action for HTE.

It has been reported that *MTP* expression is negatively modulated by SREBPs through the SRE region^34^. However, the addition of 25-HC did not alter the inhibitory effect of HTE on *MTP* and *APOB* expression in HepG2 cells (Fig. 6k). Thus, the action modes of HTE on *MTP* and *APOB* are sterol content-independent, which is different from that on *LDLR*. The *MTP* and *APOB* promoters both harbor HNF4α binding sites, which are the direct repeat 1 (DR1) region of *MTP* and the direct repeat 2 (DR2) region of *APOB*, respectively^35,36^. In an effort to identify whether HTE affects these regulatory elements, various luciferase reporter constructs were transfected into HeLa cells (Fig. 6l) followed by HTE treatment. The activities of the intact *MTP* (−165 to +30 bp) and *APOB* (−155 to +131 bp) promoters were markedly attenuated by HTE (Fig. 6m, n). The deletion or mutation of the DR1 region (TGACCTTTCCCCA→TGTGCTTTCCCCA) but not SRE region (GCAGCCCAC) abolished the inhibitory effect of HTE on *MTP* luciferase activity (Fig. 6m). Similarly, ablating the DR2 region in the *APOB* promoter (CCTTTTGCAATCCT→TTCCCCGCGGTAAT) also abrogated the reduction of *APOB* promoter activity mediated by HTE (Fig. 6n). This observation indicated that the HTE-responsive element in the *MTP* and *APOB* promoters resides in the HNF4α-binding site. In addition, the expression of two other typical HNF4α-regulating genes (*G6Pase* and *PEPCK*) was also decreased upon HTE treatment (Supplementary Fig. 6). The overexpression of HNF4α rescued the down-regulation of *MTP* and *APOB* by HTE treatment (Fig. 6o, p). Taken together, HTE decreased the expression of *MTP* and *APOB* by suppressing their regulatory transcription factor HNF4α.

Therefore, besides the NPC1L1-SREBP2-LDLR axis, another way by which HTE lowers serum cholesterol is to decrease VLDL production by inhibiting HNF4α-driven MTP and/or APOB expression.

### Hawk tea contains little caffeine and abundant flavonoids

Thus far, we concluded that hawk tea works as a cholesterol-lowering agent through a multi-target mode: promoting LDLR-mediated LDL clearance by inhibiting NPC1L1-mediated free cholesterol uptake and inhibiting VLDL production by repressing HNF4α-mediated *MTP* and *APOB* expression; in addition, gut microbiota also contributed to its cholesterol-lowering function. To identify the active components in hawk tea, we collected six batches of hawk teas and analyzed the varieties and contents of pure caffeine and polyphenols, using liquid chromatography-tandem mass spectrometry (LC-MS/MS). We also included six batches of two kinds of *Camellia* tea, green teas and black teas, for comparisons. The retention times, quantitative ion pairs, declustering potentials, collision energy, and cell exit potential of the tested compounds are listed in Supplementary Table 3.

The composition of HTE could be reliably reproduced, and the batch-to-batch variations for most components were <10% (Table 1). The caffeine content in green teas and black teas was 2542.93 ± 203.48 and 2297.13 ± 126.03 μg g^−1^, respectively (Table 1). However, there was little caffeine (35.27 ± 2.04 μg g^−1^) in hawk teas (Table 1). As mentioned above, high levels of caffeine may cause side effects like sleep deprivation and anxiety^37^. Therefore, hawk tea is superior to green or black tea in safeness for caffeine-sensitive populations.

**Table 1:** Contents of caffeine, catechins, and non-catechin flavonoids in green tea, black tea, and hawk tea

Ingredient	Green tea (*n* = 6)		Black tea (*n* = 6)		Hawk tea (*n* = 6)	
	µg g^−1^	CV (%)	µg g^−1^	CV (%)	µg g^−1^	CV (%)
Caffeine	2542.93 ± 203.48	8.0	2297.13 ± 126.03	5.49	35.27 ± 2.04	5.78
Catechin						
(+)-Catechin (C)	356.67 ± 25.98	7.28	13.95 ± 0.43	3.08	1707.85 ± 43.13	2.53
(−)-Epicatechin (EC)	3298.06 ± 76.32	2.31	602.56 ± 33.20	5.51	1024.77 ± 24.31	2.37
(−)-Catechin gallate (CG)	1840.22 ± 34.18	1.86	46.91 ± 4.27	9.10	14.90 ± 0.65	4.34
(−)-Epicatechin gallate (ECG)	2601.12 ± 77.22	2.97	427.02 ± 31.22	7.31	18.04 ± 1.02	5.66
(−)-Gallocatechin (GC)	2541.68 ± 33.61	1.32	61.54 ± 4.22	6.86	14.78 ± 0.98	6.65
(−)-Epigallocatechin (EGC)	462.37 ± 17.75	3.84	397.05 ± 17.54	4.42	34.25 ± 0.98	2.86
(−)-Gallocatechin gallate (GCG)	172.05 ± 20.11	11.69	662.28 ± 38.11	5.75	26.37 ± 1.80	6.83
(−)-Epigallocatechin gallate (EGCG)	445.64 ± 35.30	7.92	378.11 ± 26.16	6.92	35.83 ± 2.40	6.71
Non-catechin flavonoids						
Kaempferol	13.90 ± 2.33	16.78	31.87 ± 3.69	11.58	84.92 ± 3.23	3.81
Kaempferol 3-*O*-*α-*l*-*rhamnoside	1.04 ± 0.42	40.69	2.33 ± 0.15	6.37	1365.78 ± 61.81	4.53
Kaempferol 3-*O*-*β*-d-glucoside	–	–	13.04 ± 0.94	7.24	551.88 ± 25.74	4.66
Quercetin	18.42 ± 2.39	12.98	14.07 ± 1.51	10.74	92.82 ± 1.25	1.35
Quercetin 3-*O*-*β*-d-glucoside	50.92 ± 2.28	4.47	15.60 ± 1.23	7.91	516.52 ± 14.87	2.88
Quercetin 3-*O*-*β*-d-galactoside	70.37 ± 3.83	5.44	25.65 ± 5.05	19.69	664.82 ± 43.05	6.48
Quercetin 3-*O*-*α-*l-rhamnoside	0.02 ± 0.01	44.29	0.54 ± 0.18	32.78	47.05 ± 3.04	6.46
Rutin	56.24 ± 2.46	4.34	15.13 ± 1.08	7.11	45.50 ± 2.78	6.10
Apigenin	0.15 ± 0.02	14.05	0.37 ± 0.02	6.27	0.61 ± 0.03	4.37
Apigenin 3-*O*-*β*-d-glucoside	0.29 ± 0.22	75.70	0.12 ± 0.01	10.89	0.42 ± 0.04	8.98
Isorhamnetin	0.46 ± 0.03	7.05	1.54 ± 0.10	6.28	1.86 ± 0.05	2.46
Isorhamnetin 3-*O*-*β*-d-glucoside	0.20 ± 0.21	104.81	0.14 ± 0.06	41.00	1.15 ± 0.08	6.75
Naringenin	2.14 ± 1.33	62.28	9.72 ± 0.39	4.02	3.61 ± 0.27	7.54
Naringin	–	–	–	–	–	–
Luteolin	0.41 ± 0.13	30.72	0.98 ± 0.01	0.87	1.24 ± 0.06	4.83
Luteolin 7-*O*-*β*-d-glucoside	–	–	–	–	–	–
Chrysoeriol	–	–	0.20 ± 0.01	4.71	0.08 ± 0.01	16.51
Taxifolin	0.92 ± 0.10	10.64	0.61 ± 0.14	22.79	1.39 ± 0.10	7.24
Eriodictyol	0.44 ± 0.02	5.20	1.29 ± 0.03	2.24	0.14 ± 0.05	38.49
Myricetin	8.45 ± 0.35	4.18	1.88 ± 0.29	15.62	0.58 ± 0.14	24.91

– not detected, *CV* coefficient of variation

Unlike green tea and black tea, most of the polyphenols in hawk tea were non-catechin flavonoids (Table 1) and the total content of catechins in hawk tea was much lower than that in green tea and black tea (Table 1). The top six most abundant non-catechin flavonoids in hawk tea include kaempferol 3-*O*-*α*-l-rhamnoside (k-3-rha), quercetin 3-*O*-*β*-d-galactoside (q-3-gal), kaempferol 3-*O*-*β*-d-glucoside (k-3-glu), quercetin 3-*O*-*β*-d-glucoside (q-3-glu), kaempferol, and quercetin (Table 1).

### EGCG, kaempferol, and quercetin are active components of HTE

To explore the bioactive cholesterol-lowering components in hawk tea, we selected the top six types of catechins ((+)-catechin (C), (−)-epicatechin (EC), (−)-epigallocatechin gallate (EGCG), (−)-epigallocatechin (EGC), (−)-gallocatechin gallate (GCG), and (−)-epicatechin gallate (ECG)) and the top six types of non-catechin flavonoids (k-3-rha, q-3-gal, k-3-glu, q-3-glu, kaempferol, and quercetin) as candidates. The interaction of these 12 compounds with NPC1L1 or HNF4α was predicted using virtual screening with different strategies.

**Fig. 7:**
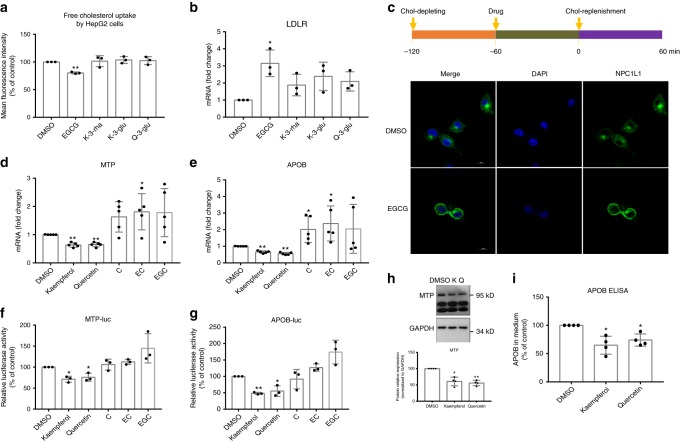
EGCG ((−)-epigallocatechin gallate), kaempferol, and quercetin are active components of hawk tea extract (HTE). **a** Free cholesterol uptake analysis (*n* = 3) and **b** quantitative real-time PCR (qRT-PCR) analysis of low-density lipoprotein receptor (*LDLR*) (*n* = 3) under the treatment of four potential Niemann–Pick C1-like 1 (NPC1L1) inhibitors (100 μM) in HepG2 cells. **c** NPC1L1 translocation analysis under EGCG treatment (50 μM) in CRL1601/NPC1L1-EGFP cells. Scale bar = 20 μm. The green fluorescence signals indicate the distribution of NPC1L1 protein. **d** qRT-PCR analysis of microsomal triglyceride transfer protein (*MTP*) and **e** apolipoprotein B (*APOB*) under the treatment of five potential hepatocyte nuclear factor 4α (HNF4α) ligands (100 μM) in HepG2 cells (*n* = 5). **f** Luciferase activity of pGL3-MTP and **g** pGL3-APOB promoter in exposure to five potential HNF4α ligands (100 μM) in HeLa cells (*n* = 3). **h** Representative Western blot analysis of MTP and quantitative analysis of MTP protein (*n* = 4) and **i** secretion of APOB under kaempferol (100 μM) and quercetin (100 μM) treatment in HepG2 cells (*n* = 4). Original Western blot images are shown as the Supplementary Fig. 16. K: kaempferol; Q: quercetin. Data are shown in mean ± SD. Statistical analyses were conducted using paired Student’s *t* test. *p < 0.05; **p < 0.01. Filled circles indicate the individual data points of each independent experiments under the indicated treatment conditions

A set of NPC1L1 inhibitors is known, but the structure of a NPC1L1-inhibitor complex has not yet been solved. Therefore, the ligand-based method, pharmacophore, was used to discover NPC1L1 inhibitors in hawk tea. The six most promising hits in hawk tea with NPC1L1 inhibitory activity include the catechins GCG, EGCG, and ECG and the flavonol glycosides q-3-glu, k-3-glu, and k-3-rha (Table 2). The matching graph of NPC1L1 pharmacophore with EGCG is shown in Supplementary Fig. 7a as an example. Next, the bioactivity of the top four hits (GCG and ECG were not included considering their low content in hawk tea leaves, <30 μg g^−1^) in HepG2 cells was evaluated at a concentration of 100 μM. Among them, only EGCG demonstrated an inhibition on free cholesterol uptake and stimulation on *LDLR* expression (Fig. 7a, b). Further, NPC1L1 translocation assays in CRL1601/NPC1L1-EGFP models revealed that EGCG inhibits the endocytosis of NPC1L1 from the plasma membrane to ERC (Fig. 7c). These results indicate that EGCG is a representative bioactive component in hawk tea, mediating its effects on free cholesterol uptake and *LDLR* expression.

**Table 2:** The Fit values of 12 candidate compounds for the screening of NPC1L1 inhibitors based on pharmacophore modeling

Name	Fit value
Ezetimibe	0.76
GCG	0.73
EGCG	0.66
ECG	0.63
Quercetin 3-*O*-*β*-d-glucoside	0.38
Kaempferol 3-*O*-*β*-d-glucoside	0.37
Kaempferol 3-*O*-*α*-l-rhamnoside	0.29
Quercetin	0
C	0
Quercetin 3-*O*-*β*-d-galactoside	0
EC	0
Kaempferol	0
EGC	0

Note that a higher Fit value means better match between the compound and the pharmacophore model

*NPC1L1* Niemann–Pick C1-like 1, *EGCG* (−)-epigallocatechin gallate, *GCG* (−)-gallocatechin gallate, *ECG* (−)-epicatechin gallate, *C* ( + )-catechin, *EC* (−)-epicatechin, *EGC* (−)-epigallocatechin

As mentioned above, HTE inhibits *MTP* and *APOB* expression through a HNF4α-dependent mechanism. HNF4α contains a putative ligand-binding domain, and the binding of antagonistic fatty acyl ligands to this domain may result in the suppression of HNF4α activity and may account for the suppression of downstream gene by these nutrients^38,39^. We next attempted to identify potential HNF4α ligands from the 12 candidates in hawk tea. Considering the limited number of known HNF4α ligands and sufficient structural information for the HNF4α ligand-binding domain, structure-based molecular docking models with the CDOCKER algorithms were employed (Supplementary Table 4). The endogenous fatty acid ligand of each HNF4α protein model was regarded as the reference compound^40^. Here, the top five compounds with relatively high -CDOCKER energy include the flavonoids kaempferol and quercetin, and the catechins C, EC, and EGC (Table 3). The 3D molecular docking results of HNF4α with kaempferol and quercetin are shown in Supplementary Fig. 7b as examples. Next, we used *MTP* and *APOB* as gene signatures to evaluate their efficacy in HepG2 cells. Among these five compounds, kaempferol and quercetin, but not the catechins C, EC, and EGC, decreased the expression of *MTP* and *APOB* at a concentration of 100 μΜ (Fig. 7d, e). The luciferase reporter assays in HeLa cells also revealed that only kaempferol and quercetin decreased *MTP* and *APOB* promoter activity, but not the catechins (Fig. 7f, g). Furthermore, we confirmed that kaempferol and quercetin decreased MTP protein amount and suppressed APOB secretion from HepG2 cells (Fig. 7h, i). These results suggest that the flavonoids kaempferol and quercetin are bioactive components that mediate the inhibitory effects of HTE on the assembly and secretion of VLDL in HepG2 cells.

**Table 3:** The -CDOCKER energy values of 12 candidate compounds for the screening of HNF4α ligands based on molecular docking

Name	−CDOCKER energy (kcal mol^−1^)		
	1PZL	1M7W	3FS1
Fatty acid	58.39	41.18	48.56
Quercetin	31.79	36.77	33.69
C	33.90	32.65	34.72
EC	35.39	35.37	28.82
Kaempferol	30.52	32.86	31.23
EGC	32.11	23.82	26.12
ECG	6.30	−2.96	−1.02
EGCG	−6.11	−7.51	−11.40
GCG	−17.76	−20.39	−15.10
Quercetin 3-*O*-*β*-d-glucoside	−85.35	−65.76	−35.31
Kaempferol 3-*O*-*β*-d-glucoside	−71.07	−61.97	−42.77
Kaempferol 3-*O*-*α*-l-rhamnoside	−65.71	−61.49	−46.81
Quercetin 3-*O*-*β*-d-galactoside	−85.00	−79.91	−64.22

Note that a higher -CDOCKER energy of compounds indicates better interaction with the ligand-binding domain of HNF4α

*NPC1L1* Niemann–Pick C1-like 1, *EGCG* (−)-epigallocatechin gallate, *GCG* (−)-gallocatechin gallate, *ECG* (−)-epicatechin gallate, *C* ( + )-catechin, *EC* (−)-epicatechin, *EGC* (−)-epigallocatechin, *HNF4α* hepatocyte nuclear factor 4α

**Fig. 8:**
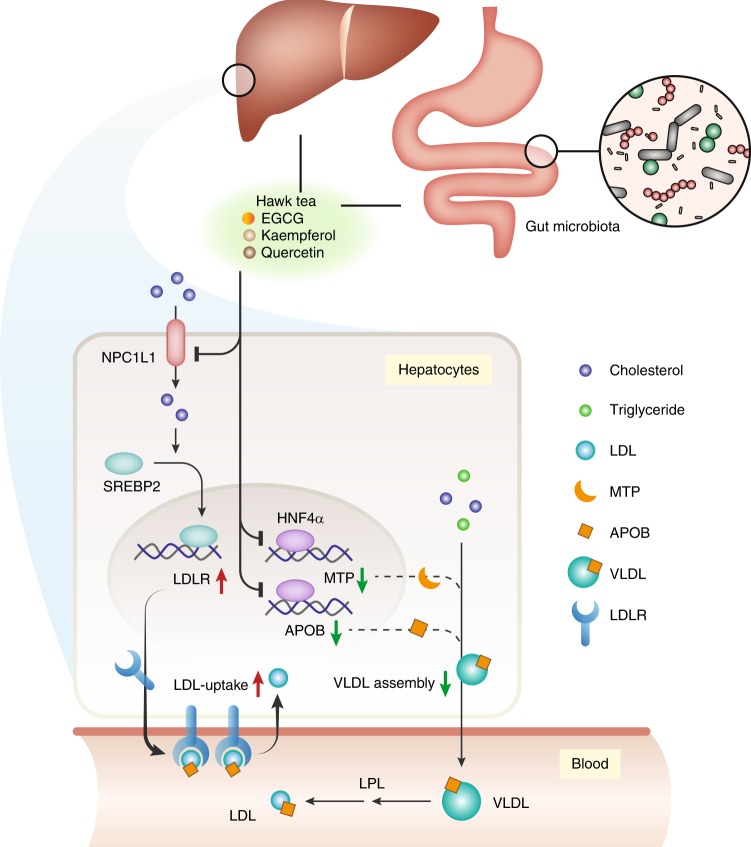
Proposed multi-target and multi-component action modes of hawk tea as a previously unrecognized cholesterol-lowering agent. In brief, hawk tea extract (HTE) inhibits Niemann–Pick C1-like 1 (NPC1L1)-mediated free cholesterol uptake by HepG2 cells, thereby inducing the transcription of low-density lipoprotein receptor (*LDLR*) downstream of the sterol response element binding protein 2 (SREBP2) pathway, and promoting the clearance of LDL. Meanwhile, HTE suppressed hepatocyte nuclear factor 4α (HNF4α)-mediated transcription of microsomal triglyceride transfer protein (*MTP*) and apolipoprotein B (*APOB*), thereby decreasing the production of very-low-density lipoprotein (VLDL) by HepG2 cells. The catechin (−)-epigallocatechin gallate (EGCG) and the flavonoids kaempferol and quercetin are bioactive components mediating the effects of HTE on the NPC1L1-SREBP2-LDLR axis and HNF4α-MTP/APOB axis in HepG2 cells. In addition, the gut microbiota serves as another target of hawk tea. LPL: lipoprotein lipase

Together, our data suggest that EGCG, kaempferol, and quercetin represent the two classes of bioactive components in hawk tea, together contributing to its cholesterol-lowering effects. Overall, hawk tea lowers cholesterol in a multi-target and multi-component manner (Fig. 8).

## Discussion

The inadequate knowledge about the molecular mechanisms and bioactive compounds of medicinal plants has become a deterrent to the worldwide acceptance of their use^9^. In the present study, using the medicinal plant hawk tea as an example, we revealed its multi-target and multi-component modes of action as a previously unrecognized cholesterol-lowering agent through the combinatorial uses of several high-throughput platforms, that is, transcriptome profiling, 16S rDNA-sequencing, and in silico virtual screening. This systematic analysis can be applied to the study of other medicinal plants.

High-throughput transcriptome profiling provides a huge amount of information on genome-wide gene disturbance caused by drugs. In our study, both in vivo and in vitro transcriptome profiling was performed and similar conclusions were drawn that HTE interfered with lipid metabolism-related pathways. Both in vivo and in vitro, HTE up-regulates the expression of *LDLR*, the most popular effector molecule to lower serum LDL-c, downstream of SREBP2 pathway. As the two classes of most commonly prescribed cholesterol-lowering drugs, Statins and Ezetimibe can both indirectly promote *LDLR* expression by directly targeting the rate-limiting enzyme in cholesterol biosynthesis, HMGCR, and the key transporter protein in free cholesterol uptake, NPC1L1, respectively. We found that HTE promotes LDLR expression in a similar manner to Ezetimibe, which rapidly inhibits cholesterol absorption by targeting NPC1L1, thereby inducing SREBP2 pathway activation and *LDLR* transcription^31^. Thus, the activation of the NPC1L1-SREBP2-LDLR axis is one of the molecular mechanisms by which HTE lowers cholesterol. To our knowledge, this is the first report of an extract from a non-*Camellia* tea acting as a type of NPC1L1 endocytosis inhibitor, suggesting that specific compounds exist in hawk tea that disrupt the recycling of NPC1L1. Considering that Ezetimibe is the only available clinical cholesterol absorption inhibitor known to target the NPC1L1 pathway^30^, the discovery of hawk tea as a previously unrecognized, free cholesterol uptake inhibitor provides a promising alternative to Ezetimibe through a dietary approach.

As two proteins essential for VLDL assembly, inhibition of MTP and/or APOB reduces the production of LDL in the circulation. Lipid-lowering drugs with these two molecules as direct targets to complement the unmet need of familial hypercholesterolemia patients by conventional therapies include the small-molecule inhibitor of MTP, Lomitapide^6^, and the antisense oligonucleotide of *APOB*, Mipomersen^7^. Different from these two drugs, we found that HTE works not by directly targeting MTP or APOB, but rather by suppressing their coordinate upstream regulator, HNF4α, suggesting the existence of potential HNF4α ligands in hawk tea. Therefore, the inhibition of the HNF4α-MTP/APOB axis is another mechanism by which HTE lowers cholesterol.

The activation of NPC1L1-SREBP2-LDLR axis leads to increased LDL clearance, while the inhibition of HNF4α-MTP/APOB axis results in decreased LDL production. The simultaneous action of HTE on these two axes mediated by different bioactive components demonstrated the advantages of medicinal plants for the treatment of complex chronic polygenic diseases such as cardiovascular diseases. Moreover, Lomitapite and Mipomersen are often accompanied by side effects, including hepatic steatosis and elevated transaminases^41^. In our study, HTE reduced the expression of MTP without causing sides effects like liver steatosis, and it even attenuated hepatic lipid accumulation in rats and lowered rat serum transaminases, indicating the superiority in safety of medicinal plants.

It is challenging to establish the direct correspondence between the pharmacological activities and chemical components of medicinal plants. Although more than 29 monomeric compounds have been identified in hawk tea^14^, the specific bioactive components are still not clear. Compared to random screening, virtual screening targeting NPC1L1 or HNF4α makes the screening of bioactive components in hawk tea more pertinent. By combining virtual screening and bioassay validation, the effect of HTE on the NPC1L1-SREBP2-LDLR axis and HNF4α-MTP/APOB axis in HepG2 cells can be reproduced by the catechin EGCG and the flavonoids kaempferol and quercetin, respectively. It has been reported that EGCG inhibits free cholesterol uptake by interfering with micellar solubilization^42^. In our study, we found evidence that EGCG inhibits free cholesterol uptake by blocking NPC1L1 endocytosis. Both kaempferol and quercetin have been reported as potential anti-hypercholesterolemic agents due to their anti-oxidant or anti-inflammatory properties^43,44^. To our knowledge, we demonstrated for the first time that kaempferol and quercetin may act as HNF4α ligands to mediate inhibitory effects on *MTP* and *APOB* expression. Taken together, it is the multiple bioactive components in hawk tea that yield its multi-target action mode.

In summary, we demonstrate that as a medicinal plant with cholesterol-lowering function, hawk tea has the following features: first, it integrates the efficacy of Ezetimibe, MTP inhibitors, and APOB inhibitors, without causing hepatotoxicity and nephrotoxicity in vivo; second, it improves the composition of the gut microbiome; third, it is safer for caffeine-sensitive populations; and last, it contains multiple bioactive components, mediating different functions. In addition, it is easily accessible in a dietary approach, and human intake of 5 cups per day (2–4 g per cup^45^) is more or less the amount we used in rats. The limitations of our study include that the mechanistic studies were mainly conducted in immortalized hepatocyte cell line (i.e., HepG2) instead of primary hepatocytes. The immortalized cell line is different from the in vivo cells from many aspects and therefore the molecular mechanisms validated in cell lines might not be fully applicable in vivo. In addition, pre-clinical and clinical studies are needed to assess the safety and efficacy of HTE in humans, though it has a long history as a folk tea. Collectively, the findings from this research support the broader application of hawk tea against metabolic disorders in the future and shed light for the use of medicinal plants in the prevention and treatment of cardiovascular diseases.

## Methods

### Materials

NBD-labeled cholesterol was obtained from Invitrogen (Carlsbad, CA, USA). *N*-Methyl-*N*-trimethylsilyl-trifluoracetamide, lathosterol, desmosterol, 25-HC, compactin, mevalonate, methyl-β-cyclodextrin, and water-soluble cholesterol were purchased from Sigma-Aldrich (St. Louis, MO, USA). Ezetimibe was purchased from Selleck Chemicals (Houston, TX, USA). Lipoprotein-deficient serum was obtained from Merck Millipore (Billerica, MA, USA). Caffeine and catechin standards were purchased from the National Institute for the Control of Pharmaceutical and Biological Products (Beijing, China). Non-catechin flavonoid standards were obtained from Baoji Herbest Bio-Tech Co., Ltd (Beijing, China). The hawk tea leaves were purchased from Yuehua Tea Group Co., Ltd (Yaan, China). The green tea (Xihu Longjing) and black tea (Qimen black tea) leaves were purchased from Beijing Hanmojuxiang Trade Center (Beijing, China).

### Cell culture

The human hepatoma cell line HepG2, human normal hepatocyte cell line HL-7702, human cervical adenocarcinoma cell line HeLa, and human colorectal adenocarcinoma cell line Caco2 were obtained from National Infrastructure of Cell Line Resource (Beijing, China). Rat CRL1601/NPC1L1-EGFP cells were generously provided by Professor Baoliang Song (School of Life Sciences, Wuhan University, China). These cells were all maintained in high glucose Dulbecco's modified Eagle’s medium (DMEM) medium supplemented with 10% fetal bovine serum and a 1% antibiotic mixture of penicillin (100 U mL^−1^) and streptomycin (100 µg mL^−1^).

Cholesterol-depleting medium was DMEM medium plus 5% lipoprotein-deficient serum, 10 μM compactin, 50 μM mevalonate, and 1.5% methyl-β-cyclodextrin. Cholesterol-replenishing medium was DMEM medium plus 5% lipoprotein-deficient serum, 10 μM compactin, 50 μM mevalonate, and water-soluble cholesterol (400 μg mL^−1^).

### Preparation of HTE

Collected hawk tea leaves were ground into powder using a grinder, weighed, and refluxed for 2 h with 90% ethanol at a mass:volume ratio of 1:30 (g mL^−1^). The extraction process was repeated twice. The final solution was then condensed under vacuum and lyophilized into a powder. Considering that there are some water-insoluble components in hawk tea ethanol extract and that dimethyl sulfoxide (DMSO) is a universal solvent for water-insoluble samples for cellular-based assays^46^, the extract was weighed and re-dissolved in DMSO to treat cells. The composition of HTE shown in Table 1 indicated the components in DMSO-dissolved contents.

### Animal studies

Male 6- to 8-week-old Sprage–Dawley rats (180–220 g) were obtained and maintained at the Beijing Vital River Laboratory Animal Technology Co., Ltd (Beijing, China). All procedures were approved by the Institutional Animal Care and Use Committee of Beijing Vital River Laboratory Animal Technology Co., Ltd. After 1 week of acclimation, animals were separated into two groups. One group was given a normal diet (*n* = 10), and the other group (*n* = 20) was given a HCHFD. The HCHFD was prepared by adding 1.2% cholesterol and 15% lard to the normal chow diet. Four weeks later, the group given the HCHFD was randomly divided into two sub-groups (*n* = 10 per group): a model group fed with an HCHFD (HCHFD group) and a group fed an HCHFD plus HTE (200 mg kg^−1^ per day) (HCHFD+HTE group). The single dose of 200 mg kg^−1^ per day was chosen because the doses of HTE in rats commonly used in previous studies are 100, 200, and 400 mg kg^−1^ per day, while 200 mg kg^−1^ per day is usually an effective dose^47–49^. This dose equals 10 g hawk tea consumption per day for a 60 kg human based on the dose conversion between rats and humans^50^, which is within the normal range of tea consumption in Chinese populations^51^. For the treatment group, HTE was dissolved in distilled water and administered via gavage every day, while an equal volume of water was used in the normal diet and HCHFD groups. Blood samples were collected, and serum lipid profiles were examined at the time points of 0, 4, 8, and 11 weeks, respectively. At the end of the 11-week treatment period, blood samples of all rats were collected after 16 h of fasting, and then all of the animals were sacrificed, except for one rat that was dropped out of the HCHFD + HTE group during the treatment period due to a possible death from acute enteritis (see Supplementary Fig. 8). Blood samples were collected, and serum was isolated after centrifugation (1200 × *g*, 10 min, 4 °C). Serum levels of total cholesterol, triglycerides, LDL-c, HDL-c, AST, ALT, BUN, and CREA were measured using kits from Roche Diagnostics (Basel, Switzerland). Livers were removed, rinsed in ice-cold phosphate-buffered saline (PBS), and prepared for RNA or protein analysis. A section from each paraffin block was stained with hematoxylin–eosin to examine the pathology of the livers, and serial cryosections were stained with Oil Red O to evaluate lipid droplets.

### 16S rDNA amplicon sequencing and bioinformatics

Fresh stool samples from each group were collected at the 0-, 4-, 8-, and 11-week time points and immediately stored at −80 °C. The genomic DNA from fecal samples was extracted using the Fast DNA Stool Extraction Kit (TIANamp, Beijing, China). DNA concentration was measured by Qubit (Invitrogen). To construct the PCR-based library for the 16S rDNA amplicon sequencing, V4-V5 dual-index fusion primer cocktail (Invitrogen) was used for PCR amplification. The PCR products were purified with AmpureXp beads (Beckman Coulter, Miami, FL, USA) to remove the non-specific products. The qualified libraries were sequenced pair end in the Miseq System (Illumina, San Diego, CA, USA) with the sequencing strategy PE300.

All pyrosequencing sequences were filtered before further analysis according to the following criteria: each sequence should contain the barcode sequences perfectly matched; a 30-bp sliding window was used to filter sequences with a lower average quality score (<20); bases with lower quality in the 5′ and 3′ of reads were trimmed; the length of the trimmed sequence shorter than 100-bp were filtered; the pair end reads that could not be merged by fastq-join were abandoned; chimera sequences identified by UCHIME were removed. All high-quality reads were aligned against the Greengenes database (version 13_5)^52^ and clustered into operational taxonomic units at the similarity level of 97% by the nearest alignment space termination algorithm. Representative sequence of each operational taxonomic unit was selected and then the taxonomical assignments of representative sequences were performed using Ribosomal Database Project classifier. Abundance of each taxonomy was calculated, and normalized for each sample for further analysis. To identify key microbes important for different treatment groups, microbe abundance was used in our statistical analysis. Prior to actual analyses, only microbes that can be detected in more than 10% of the samples and with a mean abundance more than 0.01% were included. Next, data sets involved all the three groups were assessed using one-way analysis of variance (ANOVA) followed by Tukey’s honest significant difference test. All these analyses were performed in R software (version 3.4.1.).

### RNA-seq profiling of rat livers and data analysis

The aliquots of livers from three randomly selected rats from the normal diet, HCHFD, and HCHFD+HTE groups were ground into powder in liquid nitrogen. Total RNA was extracted from using Trizol reagent (Invitrogen) according to the manufacturer’s instructions. To avoid genomic DNA contamination, total RNA samples were treated with a RNase-Free DNase Kit (Invitrogen) following the manufacturer’s instructions. The quality of RNA was evaluated using Agilent 2100 BioAnalyzer (Agilent Technologies, Palo Alto, CA, USA). The purified RNA then was used to synthesize double-strand complementary DNA (cDNA). The cDNA libraries were quantified using the Kapa Library Quantification Kit (Kapa Biosystems, Boston, MA, USA). After cluster amplification of the denatured templates, samples in flow cells were sequenced using the Illumina HiSeq X10 (Illumina) with the strategy of PE50.

StringTie (version 1.3.3b) was used to align transcript sequences obtained from RNA-seq to the UCSC reference genome rn6 and to estimate the transcript levels of genes. Differentially expressed genes were identified using DESeq2 (version 1.16.1) with the cutoff at fold change ≥2 and *p* value ≤0.05.

### CCK8 (cell counting kit-8) assay

HepG2 cells (10^4^ per well) were distributed in 96-well plates and incubated with vehicle (DMSO) or different concentrations of HTE. Cells were subjected to CCK8 reagent (Dojindo Molecular Technologies, Kumamon, Japan) at time 24 h, and incubated for another 4 h at 37 °C. Absorbance at 490 nm was read using a Spectromax spectrophotometer. The cell viability rate was calculated as: (OD experiment − OD blank)/(OD control − OD blank) × 100%.

### Gene expression profiling of HepG2 cells and data analysis

Total RNA was prepared with Trizol reagent (Invitrogen) according to the manufacturer’s instructions. To avoid genomic DNA contamination, total RNA samples were treated with a RNase-free DNase Kit (Invitrogen) following the manufacturer’s instructions.

The purified total RNA was used to produce Cy3-labeled cDNA using Agilent Low Input Quick Amp Labeling Kit (Agilent Technologies) according to the manufacturer’s instructions. Agilent SurePrint G3 Human GE 8 × 60 k microarrays were used, and the hybridization was performed according to a previous published protocol^53^. Microarrays were scanned with an Agilent’s Microarray Scanner System, and the resulting images were analyzed with the Agilent Feature Extraction software. Genes were considered differentially expressed with a 2-fold change in the treated samples compared to the control sample.

### Gene ontology and pathway analysis

To identify the functional biological processes and pathways associated with differentially expressed genes, online analytical tools such as Database for Annotation, Visualization and Integrated Discovery (DAVID)^54^ was used.

### RNA isolation, cDNA synthesis, and qRT-PCR

For HepG2, HeLa, or Caco2 cells, total RNA was isolated using a RNA Mini Kit (Qiagen, Dusseldorf, Germany) according to the manufacturer’s instructions. For liver tissues from rats, total RNA was extracted with Trizol reagent (Invitrogen). A total RNA sample (1 µg) was reverse transcribed into cDNA using a High-Capacity cDNA Reverse Transcription Kit (Thermo Fisher Scientific, Waltham, MA, USA). qRT-PCR was performed as described previously^55^. Relative mRNA levels were determined by normalizing to *GAPDH* (glyceraldehyde 3-phosphate dehydrogenase). The relative expression was calculated using the following formula: fold change = 2^−ΔΔCT^, where ΔΔCT = ΔCTsample − ΔCTcontrol; ΔCT = average CT_test gene_ − average CT_GAPDH_, and CT stands for cycle threshold. The sequences of the primer sets are available upon request.

### Construction of reporter gene plasmids for luciferase assays

The 5′-flanking promoter regions of the human *LDLR* gene (−1100 to +187 bp), *MTP* gene (−165 to +30 bp), *APOB* gene (−155 to +131 bp) were amplified from genomic DNA extracted from HEK293T cells. The PCR products were cloned into the pGL3-enhancer vector. These constructs containing the *LDLR*, *MTP*, and *APOB* promoters were designated as pGL3-LDLR, pGL3-MTP, and pGL3-APOB, respectively. Different truncation constructs were generated using specific primers. To disrupt the critical regulatory elements in their promoters, the mutant constructs were generated using a QuickChange Site-Directed Mutagenesis Kit (Stratagene, La Jolla, CA, USA) with specific primers.

For 3′-UTR activity analysis, a fragment containing the 3′-UTR of the human *LDLR* gene (2311 bp) was amplified and cloned into the *Xho*I and *Not*I restriction sites of the psiCHECK2 plasmid.

For overexpression analysis, the coding regions of human *HNF4α* were amplified from human liver cDNA and cloned into the pCMV-flag vector. The resulting plasmids encoded full-length human *HNF4α* fused to a 3× FLAG tag at the C terminus.

### Transient transfection and luciferase reporter assay

Analysis of the activity of the *LDLR*, *MTP*, and *APOB* promoters was evaluated using Promega Dual Luciferase Reporter Assay Kits (Promega) according to the manufacturer’s instructions. The firefly luciferase reporter plasmids were constructed as described above. For promoter activity analysis, a Renilla luciferase plasmid (phRL-null) without any eukaryotic promoter and enhancer elements, but still expressing a constant low amount of luciferase, was used as a control. For transfection experiments, HeLa cells were seeded into 24-well plates. On the next day, the indicated reporter plasmids with phRL-null were transfected at a ratio of 10:1 using jetPEI transfection reagent (Polyplus Transfection, Illkirch, France) following the manufacturer’s protocol. Cells were treated with vehicle or HTE for 24 h. Luciferase activity was measured with the dual luciferase assay system and a luminometer (Thermo Fisher Scientific).

### Western blot analysis

HepG2 cells, HL-7702 cells, or liver tissues were harvested and lysed in a radioimmunoprecipitation assay buffer containing 1% phenylmethylsulfonyl fluoride and protease inhibitor cocktail (Roche, Basel, Switzerland). After incubation on ice for 45 min, the lysate was centrifuged at 13,000 × *g* for 30 min at 4 °C. Then, the amount of protein in the supernatant was determined with a BCA Kit (Thermo Fisher Scientific). Cell lysates (20 µg) were separated by 10% sodium dodecyl sulfate-polyacrylamide gel electrophoresis and transferred onto polyvinylidene fluoride membranes (Millipore). The membranes were blocked in 7% (w/v) non-fat milk for 1 h at room temperature and then incubated with primary antibodies with light shaking overnight at 4 °C. Rabbit primary antibodies anti-GAPDH (1:1000, Cell Signaling Technology), anti-LDLR (1:500, Abcam, Cambridge, MA, USA), anti-SREBP2 (1:500, Abcam), anti-MTP (1:500, Abcam), anti-ERK (1:1000, Cell Signaling Technology), and anti-pERK (1:1000, Cell Signaling Technology) were used. After washing, blots were probed with goat anti-rabbit horse radish peroxidase-conjugated immunoglobulin G secondary antibody (1:5000, Cell Signaling Technology). The corresponding protein bands were visualized using enhanced chemiluminescence reagents and analyzed with a gel documentation system (Bio-Rad, Hercules, CA, USA). GAPDH was used as internal reference for each sample. The quantification of Western blot results was performed using the Image J software by normalizing the intensity of target protein bands to that of GAPDH.

### APOB ELISA

The level of APOB secreted into the medium was determined using an ELISA Kit (Abcam) according to the manufacturer’s instruction. The APOB secretion level was normalized to cellular protein content.

### Cellular and hepatic cholesterol content determination

The total cholesterol content in HepG2 cells or liver tissues was determined with a Total Cholesterol Assay Kit (Applygen Technology Inc., Beijing, China). Briefly, cellular lipids were extracted with lysis buffer, the mixture was then centrifuged (2000 × *g*, 5 min), and 10 μL of the supernatant was added to a 96-well plate containing working solution for the cholesterol assay. After incubating at 37 °C for 30 min, the absorbance at 550 nm was measured using a Varioskan Flash plate reader (Thermo Fisher Scientific). The cholesterol content was normalized to protein concentration.

### Hepatic triglycerides content determination

Hepatic triglycerides content was measured using a Triglycerides Assay Kit (Applygen Technology Inc., Beijing, China). Briefly, liver tissue (~100 mg) was homogenized in 1 mL lysis buffer in an ice-cold homogenizer. Then, the homogenate was centrifuged (2000 × *g*, 5 min), and 10 μL of the supernatant was added to a 96-well plate containing working solution for the triglycerides assay. After incubating at 37 °C for 30 min, the absorbance at 550 nm was measured using a Varioskan Flash plate reader (Thermo Fisher Scientific). The triglycerides content was normalized to protein concentration.

### Cellular lathosterol and desmosterol determination

Sample preparation was based on the method of Matysik et al.^56^ with some modifications. HepG2 cells (5 × 10^6^) were treated with drugs for 24 h, homogenized in 500 μL lysis buffer, and centrifuged (500 × *g*, 5 min). The supernatant was processed with 2 mL freshly prepared 1 M potassium hydroxide in ethanol (5.61 g in 100 mL) for 60 min at 25 °C under continuous agitation. Afterwards, the reaction solution was adjusted to pH 7.0 with phosphoric acid and 2 mL sodium chloride solution. Sterols were extracted with 2 × 3 mL and 1 × 2 mL *n*-hexane. The organic solvent was evaporated in an N2 evaporator at 35 °C and dried in a vacuum desiccator over P2O5-KOH for at least 30 min. The residue was dissolved in 50 μL of the reagent *N*-methyl-*N*-trimethylsilyltrifluoracetamide for trimethylsilylation. The derivatization reaction was performed for 60 min at 60 °C. The final solution was filtered using a 0.22 μm filter and the sample was transferred into a vial. A 1 μL aliquot was injected onto the gas chromatography.

Gas chromatography-tandem mass spectrometry was performed on a Thermo TRACE 1310 gas chromatograph equipped with a multimode inlet, a TG-5MS column (30 m, 0.25 mm, 0.25 μm df), and a Thermo TSQ-8000 mass spectrometer. Injection volume was 1 μL, the injector (270 °C) was operated in the splitless mode, and the detector transfer line was kept at 280 °C. The oven temperature program was as follows: initial temperature was 200 °C for 2 min, then to 290 °C at 45 °C min^−1^, to 320 °C at 6 °C min^−1^, and hold 5 min. Helium was used as carrier gas at 1.2 mL min^−1^. To perform the quantitative analysis, the characteristic ions and retention times of four cholesterol precursors, as reaction derivatives, were determined in the full scan (*m*/*z* 200–600), and then SRM modes were constructed to analyze them.

### Cholesterol uptake assays

HepG2 or Caco2 cells were seeded in 12-well plates and treated with HTE for indicated times on the next day. Cells were then incubated with 20 μg mL^−1^ NBD-labeled cholesterol (Invitrogen) for an additional indicated time in the dark. Cells exposed to NBD-labeled cholesterol were washed with PBS and harvested by trypsinization for fluorescence-activated cell sorting analysis (excitation: 485 nm; emission: 535 nm). The mean fluorescence intensity (MFI) of control cells was defined as 100%, and the MFIs of cells under drug treatments are expressed as the percentage of the control.

### Confocal imaging

For imaging studies, CRL1601/NPC1L1-EGFP cells were washed and fixed with 4% formaldehyde for 10 min. Cells were incubated with 4′,6-diamidino-2-phenylindole for nuclear staining. Images of NPC1L1-EGFP were captured using Nikon inverted microscope (Nikon Instruments Inc., Tokyo, Japan). Three independent experiments were performed, and one representative picture is shown in the figures.

### LC-MS/MS analyses

For the preparation of analytes, dry tea leaves were accurately weighed and sonicated in 80% methanol at a ratio of 1:30 (w/v) for 40 min using a SB-800 DTD sonicator (Ningbo Xinzhi Biotechnology Co., Ltd, Ningbo, China; Power: 100 W; Frequency: 40 kHz). The resulting supernatant was filtered through a 0.22 μm filter (PALL, Ann Arbor, MI, USA) for subsequent LC-MS/MS analysis.

LC analysis was performed on an ACQUITY UPLC^TM^ I-Class system (Waters, Milford, MA, USA) equipped with an online vacuum degasser, a binary pump, an autosampler, and a thermostat column compartment. ACQUITY UPLC BEH C18 column (100 mm × 2.1 mm i.d., 1.8 μm) was used for separation. The binary gradient of water containing 0.1% formic acid (A) and acetonitrile containing 0.1% formic acid (B) at a constant flow rate of 0.6 mL min^−1^ was applied with an injection volume of 1.0 μL. Column oven temperature was at 40 °C. The linear gradient conditions were optimized as follows: 0 min, 5% B; 2 min, 25% B; 3.5 min 40% B; 5 min 60% B.

An AB Sciex API 6500 triple quadrupole mass spectrometer with an electrospray ion source (Applied Biosystems, Toronto, Canada) was used. The MS spectra were acquired in negative ion mode (except caffeine in positive ion mode), which was carried out by optimization of the product ion obtained from the fragment of the isolated precursor ion for each analyte. The ion spray potential was 4500 V, and the source temperature was set at 550 °C. Once the product ions were chosen, the MRM (multiple reaction monitoring) conditions for each standard were further optimized to achieve maximum sensitivity.

### Virtual screening of NPC1L1 inhibitors

Pharmacophore-based virtual screening was utilized to identify the bioactive components in hawk tea with NPC1L1 inhibitory activity. First, low-energy three-dimensional conformations of 12 candidate components from hawk tea were constructed and minimized within BEST mode and the CHARMm force field by Discovery Studio^57^, and the relative energy threshold was set as <20.0 kcal mol^−1^. The virtual screening was then implemented using pharmacophore models constructed by Huo et al.^58^. With Ezetimibe as a reference, potential NPC1L1 inhibitors from hawk tea were identified for further bioactivity validation.

### Virtual screening of HNF4α ligands

Three crystal structures of HNF4α ligand-binding domain in complex with the ligands (1M7W, 1PZL, and 3FS1) from Protein Data Bank were utilized to identify potential HNF4α ligands from hawk tea based on molecular docking by CDOCKER algorithms^59^. Water molecules were cleaned from these three protein structures and hydrogen atoms were then added to the structures. The binding sites of proteins were determined by the endogenous fatty acid ligands, which were re-docked into the binding sites to evaluate the docking models by computing the root mean-square deviation (RMSD) and -CDOCKER energy. Three docking models are shown in Supplementary Table 4 and RMSDs of these models were all <2.00 Å. According to -CDOCKER energy computed by three groups of molecular docking, potential HNF4α ligands from hawk tea were identified for further bioactivity validation.

### Statistical analysis

Statistical analyses of experimental data were conducted using Student’s *t* test or the one-way ANOVA test. Values are expressed as the mean ± standard deviation or mean ± standard error of mean. A *p* value <0.05 was accepted as statistically significant.

### Reporting summary

Further information on experimental design is available in the Nature Research Reporting Summary linked to this article.

## Supplementary information

Description of supplementary data 1

Supplementary Information

Reporting Summary

Supplementary Data 1

## Data Availability

The RNA sequencing and microarray data has been deposited in Gene Expression Omnibus database under accession number [GSE125084] and [GSE117583], respectively. Source data underlying the graphs presented in the main figures are available in Supplementary Data 1. The LC-MS/MS data for the tested compounds are provided in Supplementary Table 3.
