# Supplementary material for: Caffeine-free hawk tea lowers cholesterol by reducing free cholesterol uptake and the production of very-low-density lipoprotein
Source: Commun Biol. 2019 May 8;2:173. doi: 10.1038/s42003-019-0396-4 (PMC6506518; doi:10.1038/s42003-019-0396-4)
Supplement: Supplementary file 1 — Description of supplementary data 1 [file 42003_2019_396_MOESM1_ESM.docx]

**Description of Additional Supplementary Files**

**File name:** Supplementary Data 1

**Description:** Source data underlying the graphs presented in Fig. 1a,1b,1e,2a-c,3a-h,4a,4b,4d,4f,4h,4i,4j,5a,5b,5d,5e,6a-k,6m-p,7a,7b,7d,7e,7f,7g,7i
