# Supplementary material for: Caffeine-free hawk tea lowers cholesterol by reducing free cholesterol uptake and the production of very-low-density lipoprotein
Source: Commun Biol. 2019 May 8;2:173. doi: 10.1038/s42003-019-0396-4 (PMC6506518; doi:10.1038/s42003-019-0396-4)
Supplement: Supplementary file 2 — Supplementary Information [file 42003_2019_396_MOESM2_ESM.pdf]

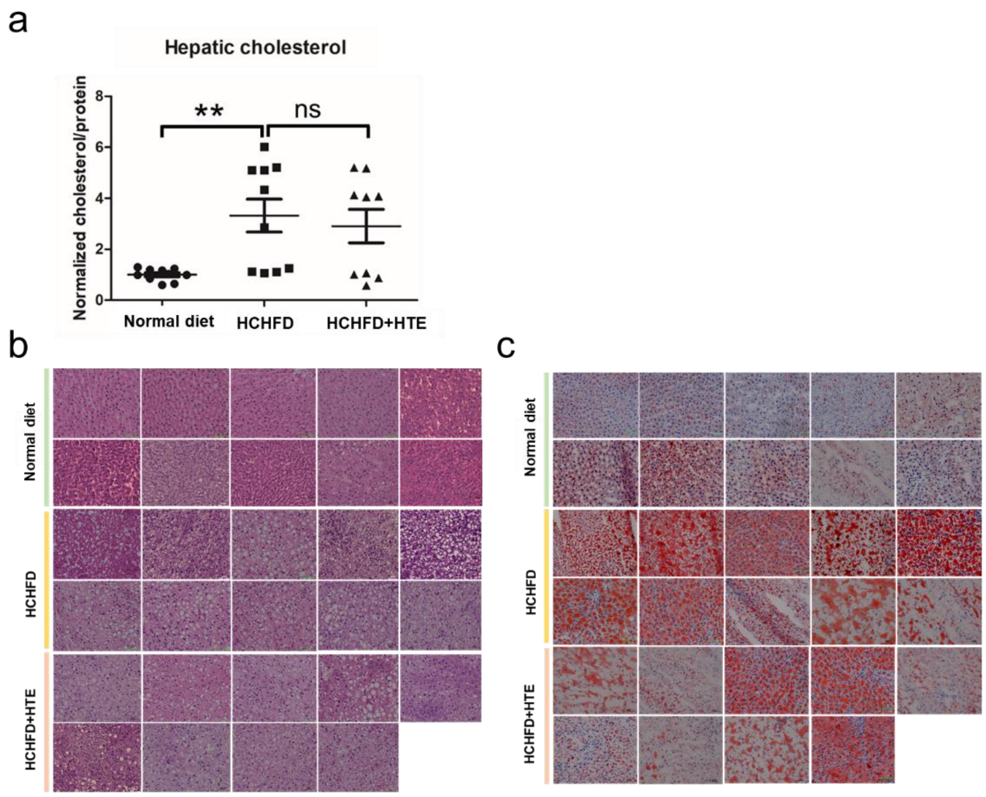

d

	Normal diet (n=10)	HCHFD (n=10)	HCHFD+HTE (n=9)
BUN (mmol/L)	6.03 ± 0.62	4.23 ± 0.88 ##	6.16 ± 0.99 **
CREA (μmol/L)	27.33 ± 2.00	27.70 ± 4.40	29.11 ± 2.03

Supplementary Figure 1. HTE ameliorates HCHFD-induced hypercholesterolemia and liver steatosis in rats. **(a)** Hepatic cholesterol levels in rats of three groups. **(b)** Hematoxylin-eosin staining and **(c)** Oil Red O staining for all the liver specimens in three groups. **(d)** Safety-related serum parameters in rats. Statistical analyses were conducted using non-paired Student's *t*-test. For **(a)**, data are shown in mean±SEM. $^{**}p < 0.01$; ns: not significant. For **(d)**, data are shown in mean±SD. $^{\#\#}p < 0.01$ vs normal diet group; $^{**}p < 0.01$ vs HCHFD group.

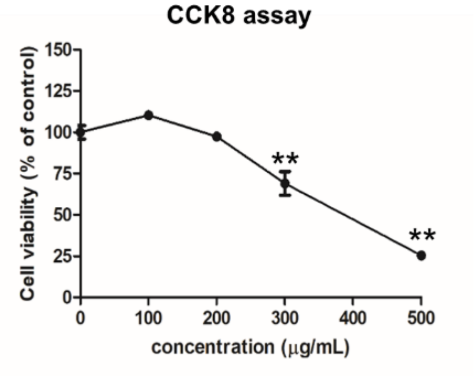

Supplementary Figure 2. Cell viability of HepG2 cells under HTE treatment determined by CCK8 assay (n=3). Data are shown in mean ± SD. Statistical analyses were conducted using paired Student's *t*-test. **p < 0.01.

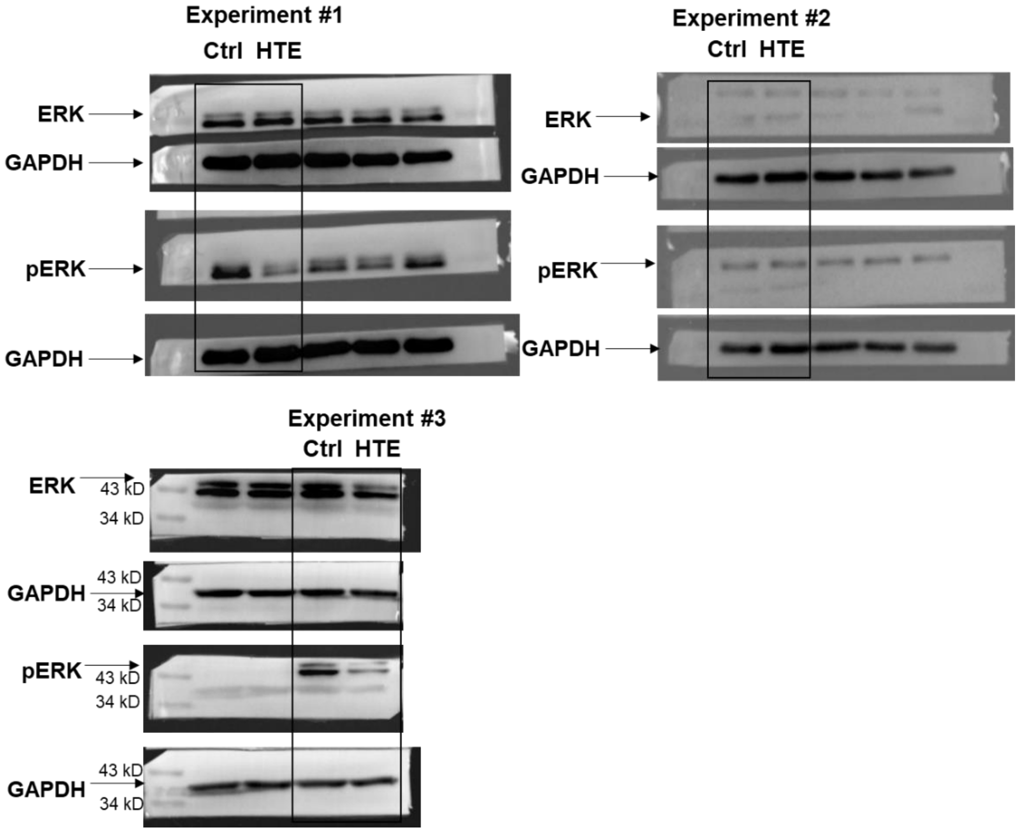

Supplementary Figure 3. Western blot analysis of total ERK and pERK in response to HTE (200 μg mL^{-1}) treatment in HepG2 cells.

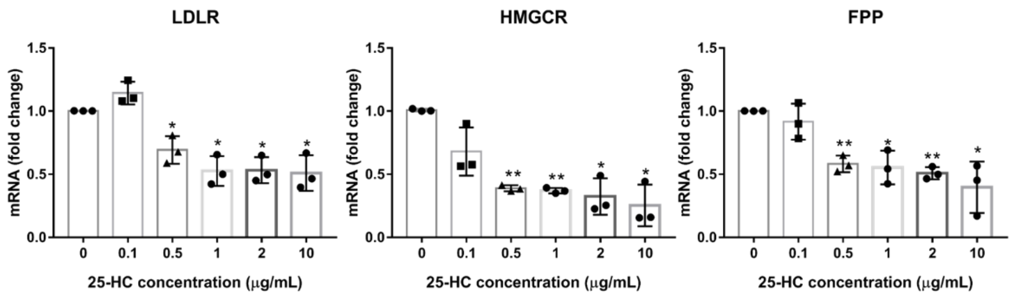

Supplementary Figure 4. Dose-response profiles of SREBP2-regulated genes expression under 25-HC treatment in HepG2 cells (n=3). Data are shown in mean ± SD. Statistical analyses were conducted using paired Student's *t*-test.*p < 0.05; **p < 0.01.

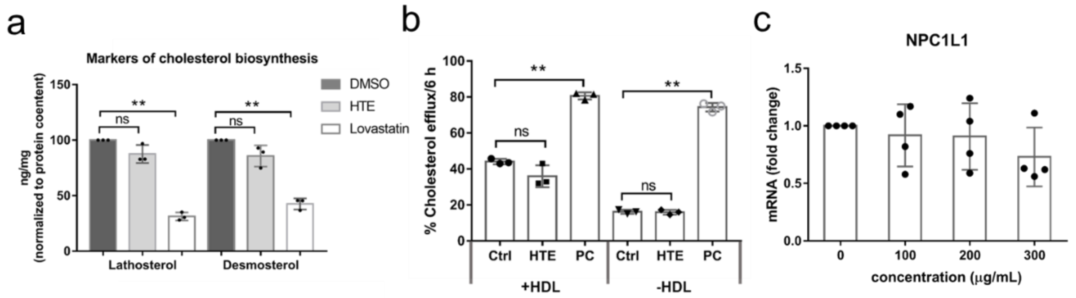

Supplementary Figure 5. HTE lowers cellular cholesterol not through affecting cholesterol biosynthesis or efflux, but by inhibiting free cholesterol uptake. **(a)** The effect of HTE (200 μg mL^{-1}) or Lovastatin (2 μM) on cholesterol biosynthesis in HepG2 cells (n=3). **(b)** Effects of HTE (200 μg mL^{-1}) on cholesterol efflux in HepG2 cells. The cells were pre-treated with fluorescently labeled cholesterol for 16 h, and then the HDL acceptor and HTE (200 μg mL^{-1}) were added to the cells for another 6 h. The level of cholesterol efflux was examined by measuring the percentage of the fluorescence intensity in the medium. The value was normalized to the cell number in each well. PC: positive control, a drug provided by the manufacturer to promote cholesterol efflux. **(c)** Dose-response profiles of *NPC1L1* expression under HTE treatment in Caco2 cells (n=4). Data are shown in mean ± SD. Statistical analyses were conducted using paired Student's *t*-test.**p < 0.01; ns: not significant.

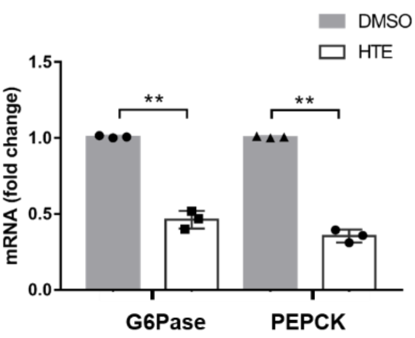

Supplemental Figure 6. qRT-PCR analysis of HNF4α-downstream *G6Pase* and *PEPCK* under HTE treatment in HepG2 cells. Data are shown in mean ± SD. Statistical analyses were conducted using paired Student's *t*-test.**p < 0.01.

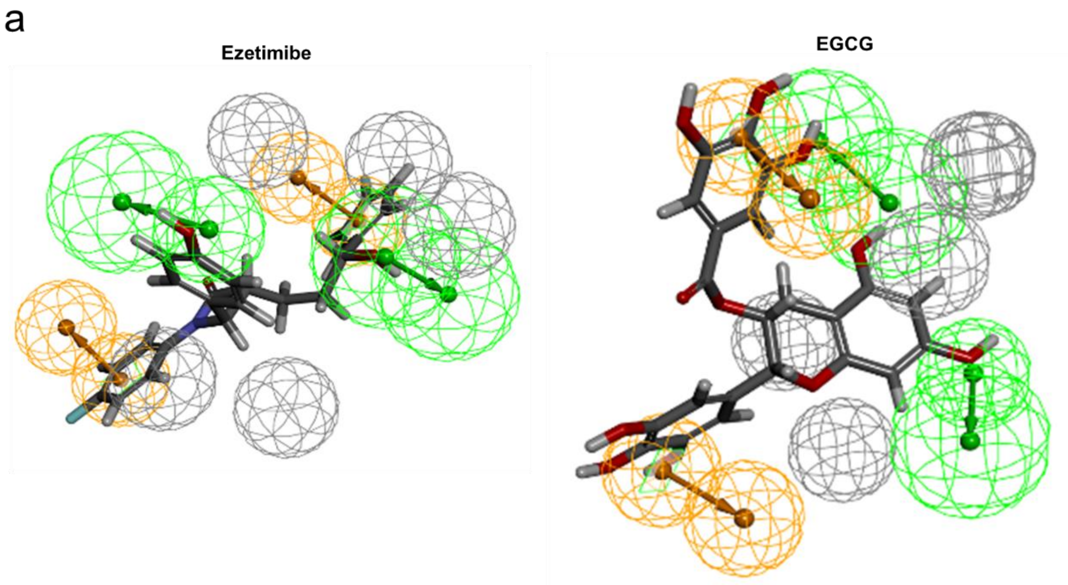

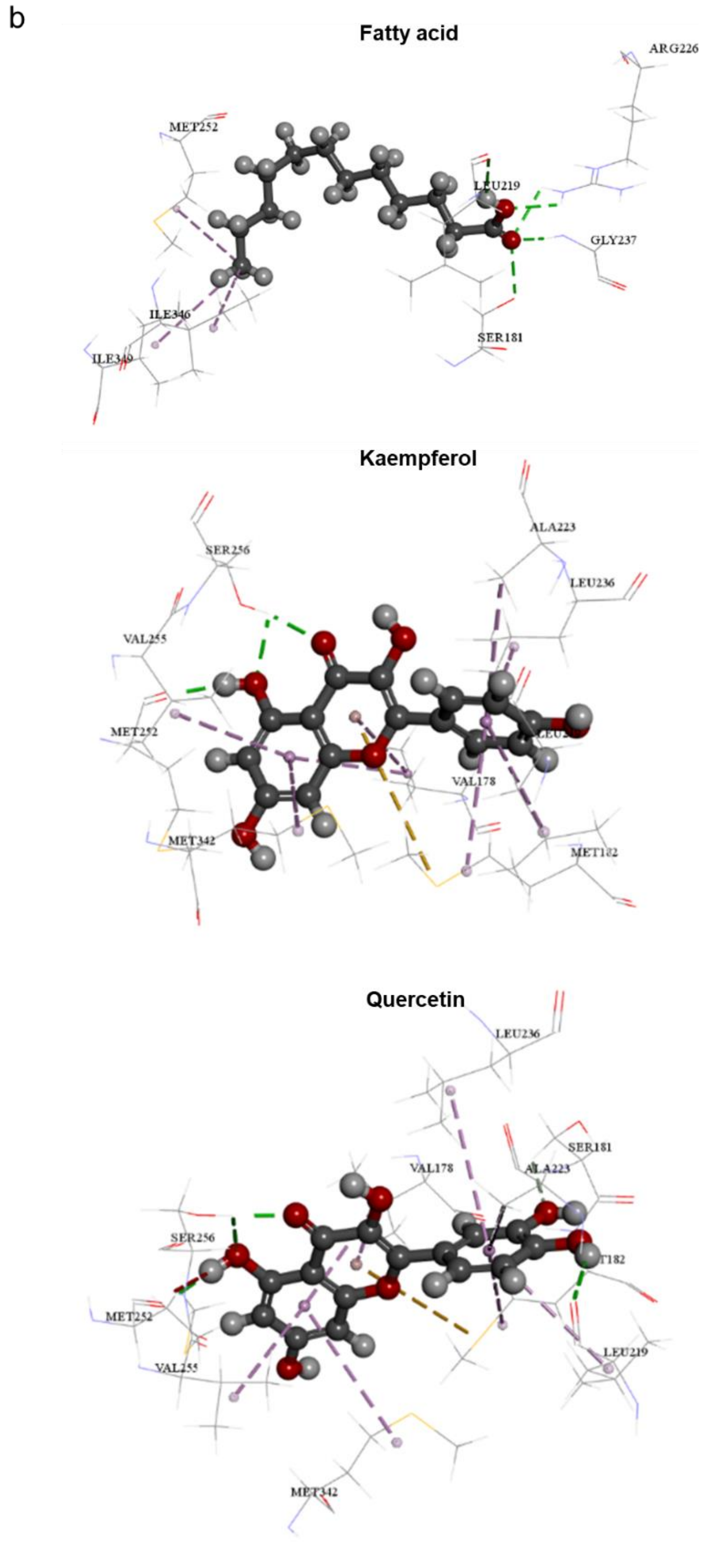

Supplementary Figure 7. EGCG, kaempferol, and quercetin are active compounds of HTE. **(a)** Matching graphs of NPC1L1 pharmacophore with Ezetimibe and EGCG. **(b)** The 3D docking results of HNF4α (1M7W) with fatty acid (lauric acid), kaempferol, and quercetin.

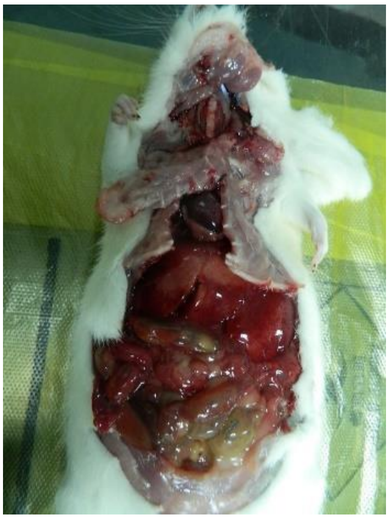

Supplementary Figure 8. The anatomy view of the rat in HTE+HCHFD group who died accidently during the treatment period.

Fig. 4c

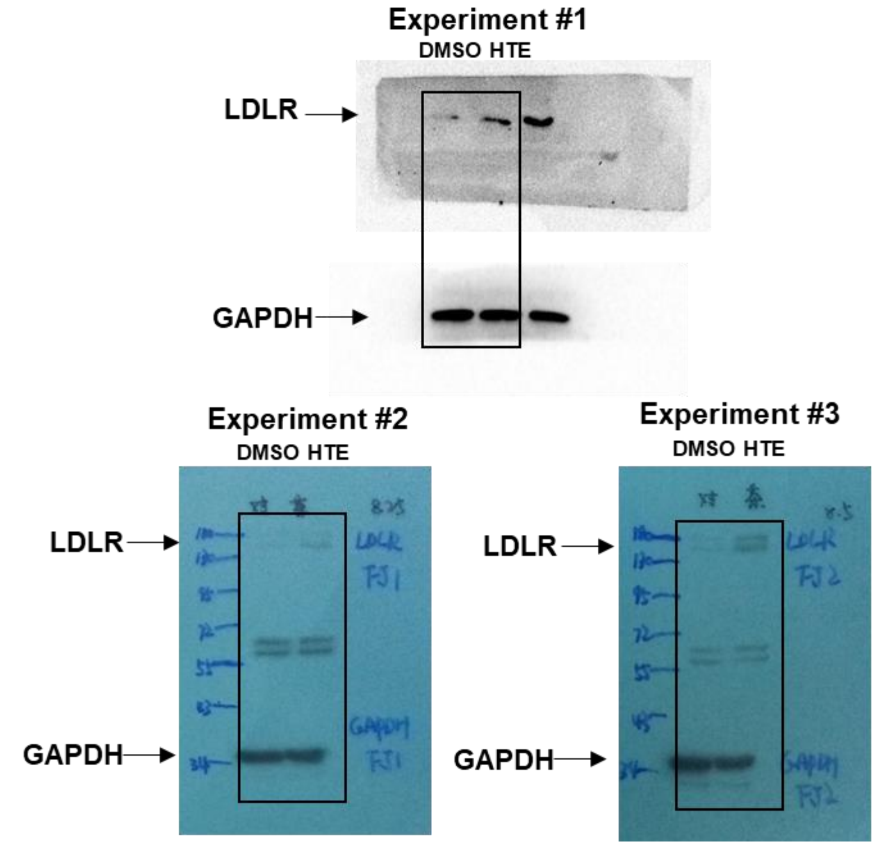

Supplementary Figure 9. The original Western blot images of Figure 4c.

Fig. 4e

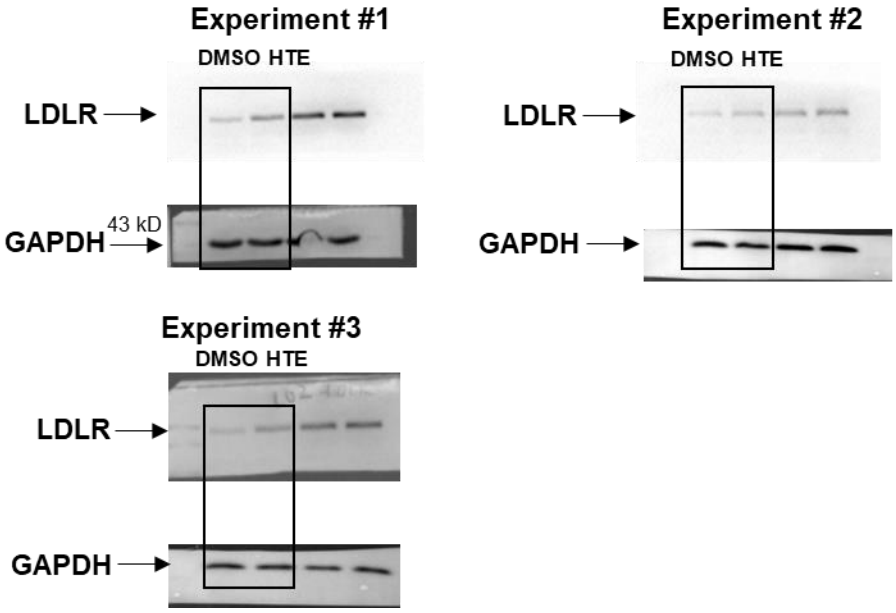

Supplementary Figure 10. The original Western blot images of Figure 4e.

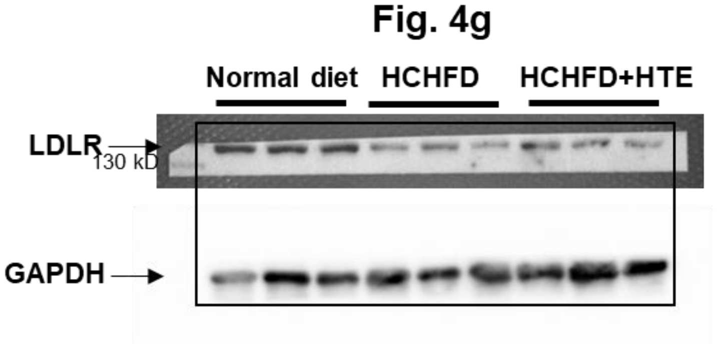

Supplementary Figure 11. The original Western blot images of Figure 4g.

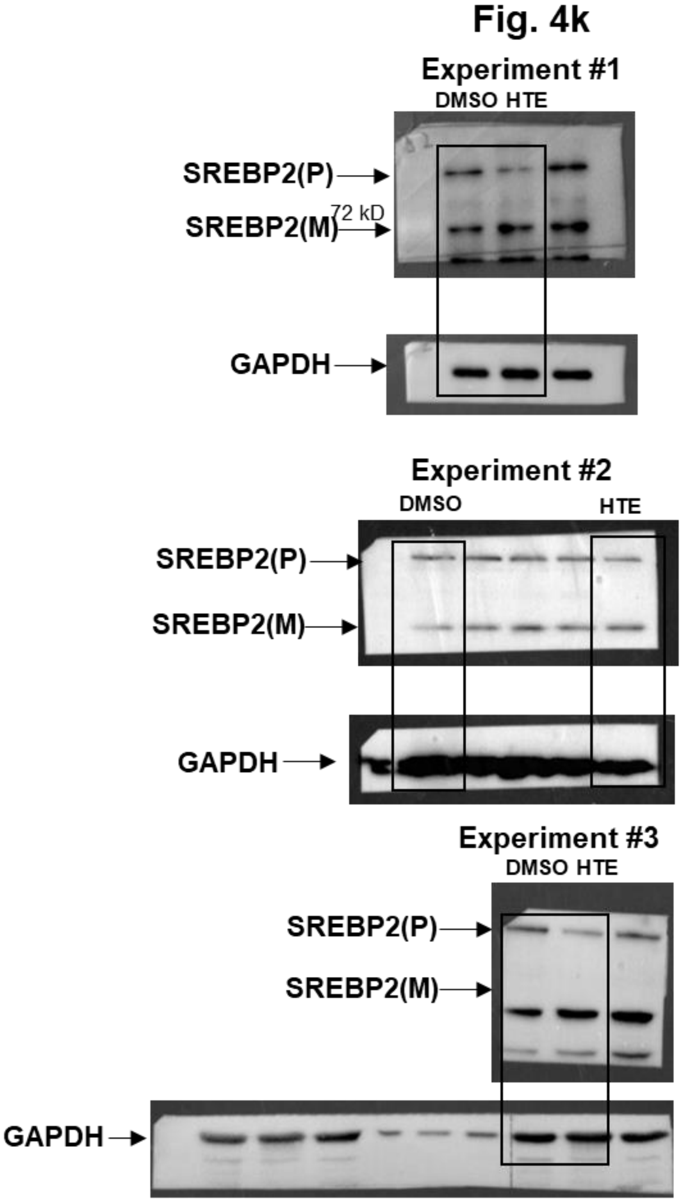

Supplementary Figure 12. The original Western blot images of Figure 4k.

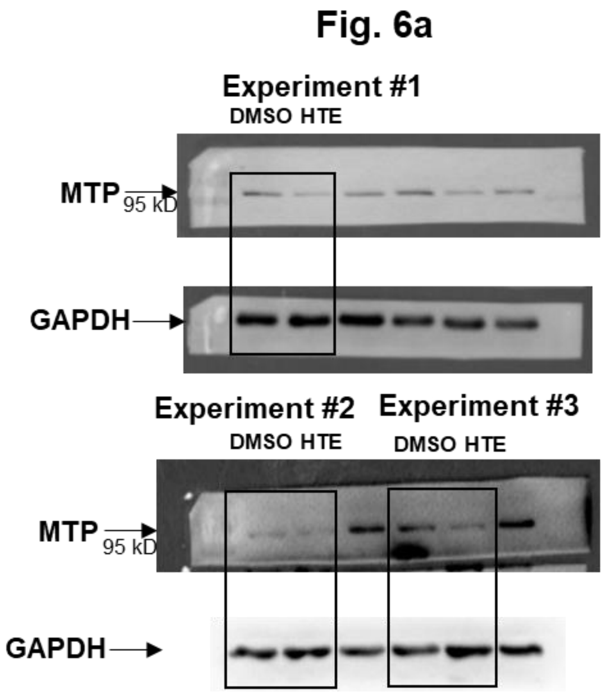

Supplementary Figure 13. The original Western blot images of Figure 6a.

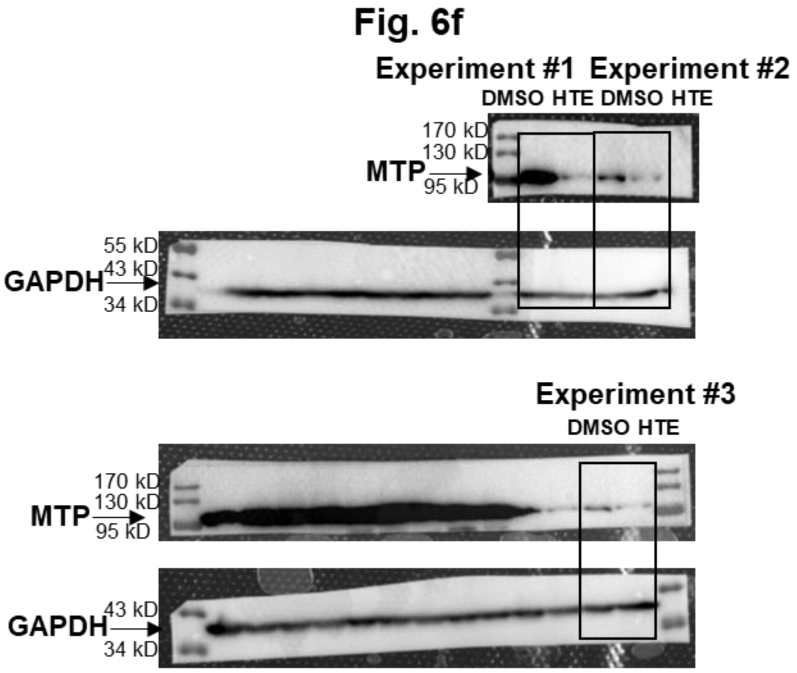

Supplementary Figure 14. The original Western blot images of Figure 6f.

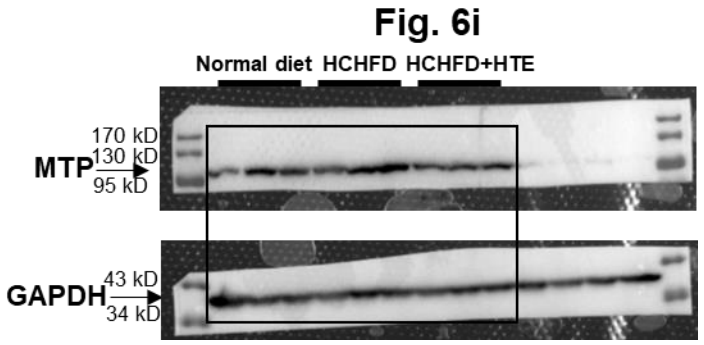

Supplementary Figure 15. The original Western blot images of Figure 6i.

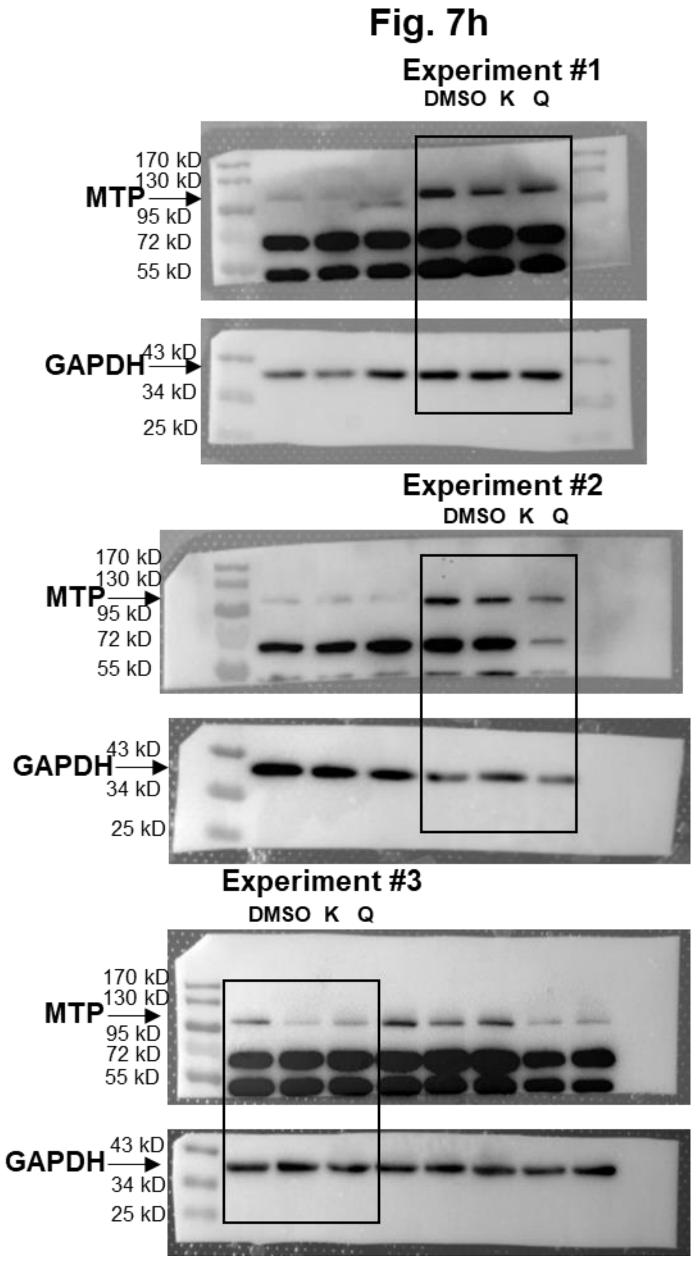

Supplementary Figure 16. The original Western blot images of Figure 7h.

Supplementary Table 1. Correlation analysis between serum total cholesterol levels and microbe abundance.

Genera	Pearson's *R* value	p value
g__Parabacteroides	0.692	0
g__Dorea	0.639	0
g__Bacteroides	0.631	0
f__Clostridiaceae	0.616	2.34E-13
g__Bilophila	0.610	4.40E-13
g__f__Erysipelotrichaceae	0.603	0
g__Roseburia	0.582	0
g__Sutterella	0.58	0
g__f__Lachnospiraceae	0.578	0
g__Prevotella	0.554	5.02E-11
g__[Eubacterium]	0.532	9.72E-10
g__f__Desulfovibrionaceae	0.524	3.04E-09
g__SMB53	0.523	3.30E-09
g__f__Peptostreptococcaceae	0.506	8.00E-09
g__[Ruminococcus]	0.503	1.66E-08
g__Lachnospira	-0.506	1.05E-08
g__Elusimicrobium	-0.545	3.08E-10
g__f__o__Bacteroidales	-0.565	4.70E-11
g__Ruminococcus	-0.596	0
g__f__o__RF39	-0.622	1.18E-13

Supplementary Table 2. Correlation analysis between serum LDL-c levels and microbe abundance.

Genera	Pearson's *R* value	p value
g__Parabacteroides	0.637	0
g__Bacteroides	0.613	0
g__Roseburia	0.578	0
g__Bilophila	0.578	1.40E-11
g__f__Desulfovibrionaceae	0.562	0
g__Lachnospira	-0.516	3.50E-09
g__Prevotella	-0.529	1.88E-09
g__f__o__RF39	-0.547	2.58E-10
g__f__o__Bacteroidales	-0.56	7.76E-11

Supplementary Table 3. Retention times (t_R), quantitative ion pairs, declustering potentials (DP), collision energy (CE), and cell exit potential (CXP) for LC-MS/MS analyses.

Analyte	t_R (min)	Quantitative ion pairs *m/z*	DP / V	CE / V	CXP / V
Caffeine	1.79	195.2/163.1	80	18	12
(+)-Catechin (C)	1.22	288.9/202.9	-79.9	-27	-10
(-)-Epicatechin (EC)	1.68	289.1/203.0	-125	-27	-11
(-)-Catechingallate (CG)	2.05	441.1/289.1	-197	-25	-13
(-)-Epicatechingallate (ECG)	2.00	441.0/289.0	-150	-27	-11
(-)-Gallocatechin (GC)	0.86	304.0/218.9	-175	-32	-15
(-)-Epigallocatechin (EGC)	1.06	304.8/124.8	-147	-34	-25
(-)-Gallocatechingallate (GCG)	1.85	457.1/305.0	-117	-20	-23
(-)-Epigallocatechingallate (EGCG)	1.71	457.0/169.1	-194	-24	-8
Kaempferol	2.89	285.0/210.7	-30	-46	-23
Kaempferol 3-*O*-*α*-L-rhamnoside	1.85	431.1/285.0	-122	-27	-15
Kaempferol 3-*O*-*β*-D-glucoside	1.52	447.1/254.8	-170	-46	-23
Quercetin	2.29	301.0/159.9	-82	-53	-19
Quercetin 3-*O*-*β*-D-glucoside	1.48	463.1/300.0	-191	-36	-22
Quercetin 3-*O*-*β*-D-galactoside	1.45	463.1/300.0	-165	-35	-19
Quercetin 3-*O*-*α*-L-rhamnoside	1.64	447.1/300.0	-97	-34	-36
Rutin	1.40	609.1/300.0	-245	-48	-38
Apigenin	2.76	269.1/117.0	-1717	-43	-19
Apigenin 7-*O*-*β*-D-glucoside	1.69	431.1/268.0	-265	-47	-40
Isorhamnetin	3.01	315.0/299.8	-25	-29	-22
Isorhamnetin 3-*O*-*β*-D-glucoside	1.66	477.0/313.9	-140	-37	-21
Naringenin	2.77	271.0/150.9	-85	-25	-17
Naringin	1.67	579.2/271.0	-217	-42	-8
Luteolin	2.26	285.0/133.0	-144	-40	-15

Luteolin 7-*O*-*β*-D-glucoside	1.51	447.0/285.0	-146	-34	-25
Chrysoeriol	2.92	299.1/284.0	-160	-27	-33
Taxifolin	1.59	302.8/285.0	-152	-19	-9
Eriodictyol	2.23	-287.0/151.0	-71	-21	-12
Myricetin	1.46	463.0/316.0	-214	-37	-28

Supplementary Table 4. The docking models for HNF4α ligand screening.

PDB code	RMSD(Å)	Radius(Å)	−CDOCKER energy (kcal/mol)	−CDOCKER interaction energy (kcal/mol)
1PZL	1.54	7.77	58.40	57.86
3FS1	1.42	6.97	48.56	48.82
1M7W (A chain)	1.17	7.25	41.18	40.67
