# Supplementary material for: Caffeine-free hawk tea lowers cholesterol by reducing free cholesterol uptake and the production of very-low-density lipoprotein
Source: Commun Biol. 2019 May 8;2:173. doi: 10.1038/s42003-019-0396-4 (PMC6506518; doi:10.1038/s42003-019-0396-4)
Supplement: Supplementary file 3 — Reporting Summary [file 42003_2019_396_MOESM3_ESM.pdf]

nature research

Corresponding author(s): Lan Xie

Reporting Summary

Nature Research wishes to improve the reproducibility of the work that we publish. This form provides structure for consistency and transparency in reporting. For further information on Nature Research policies, see Authors & Referees and the Editorial Policy Checklist.

Statistical parameters

When statistical analyses are reported, confirm that the following items are present in the relevant location (e.g. figure legend, table legend, main text, or Methods section).

n/a	Confirmed	
☐	☒	The exact sample size (*n*) for each experimental group/condition, given as a discrete number and unit of measurement
☐	☒	An indication of whether measurements were taken from distinct samples or whether the same sample was measured repeatedly
☐	☒	The statistical test(s) used AND whether they are one- or two-sided<br>*Only common tests should be described solely by name; describe more complex techniques in the Methods section.*
☒	☐	A description of all covariates tested
☐	☒	A description of any assumptions or corrections, such as tests of normality and adjustment for multiple comparisons
☒	☐	A full description of the statistics including central tendency (e.g. means) or other basic estimates (e.g. regression coefficient) AND variation (e.g. standard deviation) or associated estimates of uncertainty (e.g. confidence intervals)
☒	☐	For null hypothesis testing, the test statistic (e.g. *F*, *t*, *r*) with confidence intervals, effect sizes, degrees of freedom and *P* value noted<br>*Give P values as exact values whenever suitable.*
☒	☐	For Bayesian analysis, information on the choice of priors and Markov chain Monte Carlo settings
☒	☐	For hierarchical and complex designs, identification of the appropriate level for tests and full reporting of outcomes
☒	☐	Estimates of effect sizes (e.g. Cohen's *d*, Pearson's *r*), indicating how they were calculated
☐	☒	Clearly defined error bars<br>*State explicitly what error bars represent (e.g. SD, SE, CI)*

Our web collection on statistics for biologists may be useful.

Software and code

Policy information about availability of computer code

Data collection	No code was involved in this study.
Data analysis	DAVID (version 6.8) was used for gene ontology and pathway analysis of microarray data and RNA sequencing data. R software (version 3.4.1) was used for 16S rDNA sequencing result analysis. Image J (version 1.5) was used for Western blot quantification. Discover Studio software was used for virtual screening.

For manuscripts utilizing custom algorithms or software that are central to the research but not yet described in published literature, software must be made available to editors/reviewers upon request. We strongly encourage code deposition in a community repository (e.g. GitHub). See the Nature Research guidelines for submitting code & software for further information.

Data

Policy information about availability of data

All manuscripts must include a data availability statement. This statement should provide the following information, where applicable:

- Accession codes, unique identifiers, or web links for publicly available datasets
- A list of figures that have associated raw data
- A description of any restrictions on data availability

The RNA sequencing and microarray data has been deposited in Gene Expression Omnibus under accession number [GSE125084] and [GSE117583], respectively.

Field-specific reporting

Please select the best fit for your research. If you are not sure, read the appropriate sections before making your selection.

☒ Life sciences ☐ Behavioural & social sciences ☐ Ecological, evolutionary & environmental sciences

For a reference copy of the document with all sections, see nature.com/authors/policies/ReportingSummary-flat.pdf

Life sciences study design

All studies must disclose on these points even when the disclosure is negative.

Sample size	Usually, to calculate p value and achieve more confident results, more than 10 samples of each group were used for animal experiments. For example, 10 rats of each control group and experimental group were analyzed in our study.
Data exclusions	One rat was dropped out from the study and we discussed the case in the "Materials and Methods" section.
Replication	All experimental findings were reproduced independently for at least three times.
Randomization	Samples such as rats were randomly allocated into experimental groups.
Blinding	Investigators were not blinded to group allocation.

Reporting for specific materials, systems and methods

Materials & experimental systems

n/a	Involved in the study	
☒	☐	Unique biological materials
☐	☒	Antibodies
☐	☒	Eukaryotic cell lines
☒	☐	Palaeontology
☐	☒	Animals and other organisms
☒	☐	Human research participants

Methods

n/a	Involved in the study	
☒	☐	ChIP-seq
☐	☒	Flow cytometry
☒	☐	MRI-based neuroimaging

Antibodies

Antibodies used	Anti-LDLR antibody (ab30532; Abcam);<br>Anti-SREBP2 antibody (ab30682; Abcam);<br>Anti-MTP antibody (ab63467; Abcam);<br>Anti-p44/42 MAPK (ERK1/2): (4695; Cell Signaling Technology);<br>Anti-Phospho-p44/42 MAPK (ERK1/2): (4370; Cell Signaling Technology);<br>Anti-GAPDH antibody (2118; Cell Signaling Technology);<br>Anti-Rabbit IgG (7074; Cell Signaling Technology)
Validation	All antibodies are validated for the species and assay used as described in the manufacturer's web page.

Eukaryotic cell lines

Policy information about cell lines

Cell line source(s)	The human hepatoma cell line HepG2, human normal hepatocyte cell line HL-7702, human cervical adenocarcinoma cell line HeLa, and human colorectal adenocarcinoma cell line Caco2 were purchased from National Infrastructure of Cell Line Resource (Beijing, China). Rat CRL1601/NPC1L1-EGFP cells were generously provided by Professor Baoliang Song (School of Life Sciences, Wuhan University, China).
Authentication	All commercial cell lines were authenticated by Short Tandem Repeat (STR) analysis. Briefly,<br>1).Genomic DNA was extracted from the cell pellets.<br>2).Samples, together with positive and negative control were amplified using MicroreaderTM 21 Direct ID System kit (Microread,10401105).

	3). Amplified products were processed using the ABI3500 Genetic Analyzer system (Thermo Fisher, 4406017).<br>4).Data were analyzed using GeneMapper4.0 software and then compared with the ATCC databases for reference matching.<br>Cells provided by Professor Song were validated by published reference, i.e., Cell Metab. 2008 Jun;7(6):508-19.
Mycoplasma contamination	All cell lines were all tested negative for mycoplasma contamination.
Commonly misidentified lines<br>(See ICLAC register)	No commonly misidentified cell lines were used.

Animals and other organisms

Policy information about studies involving animals; ARRIVE guidelines recommended for reporting animal research

Laboratory animals	Male Sprage-Dawley rats, age 6-8 weeks
Wild animals	This study did not involve wild animals.
Field-collected samples	This study did not involve field-collected sample.

Flow Cytometry

Plots

Confirm that:

☒ The axis labels state the marker and fluorochrome used (e.g. CD4-FITC).

☒ The axis scales are clearly visible. Include numbers along axes only for bottom left plot of group (a 'group' is an analysis of identical markers).

☒ All plots are contour plots with outliers or pseudocolor plots.

☒ A numerical value for number of cells or percentage (with statistics) is provided.

Methodology

Sample preparation	Please refer to "Supplementary Information", section "Cholesterol absorption assay"
Instrument	A BD FASCCalibur flow cytometer was used for data collection.
Software	Cellquest and FlowJo-ModFit softwares were used to collect and analyze flow cytometry data. Further data analysis was performed using the Microsoft Excel software (version 2016).
Cell population abundance	Flow cytometry was used for quantification only and no post-sorting fractions were collected.
Gating strategy	For all experiments, FSC-A/SSC-A gates of the starting cell population were used to discriminate between viable cells and cell debris. For fluorescence signaling analysis, cells not labeled with fluorohores were used as negative controls.

☐ Tick this box to confirm that a figure exemplifying the gating strategy is provided in the Supplementary Information.
